# Prediction and experimental validation of solid solutions and isopolymorphs of cytosine/5-flucytosine†

**DOI:** 10.1039/C7CE00939A

**Published:** 2017-06-12

**Authors:** D. E. Braun, U. J. Griesser

**Affiliations:** Institute of Pharmacy, University of Innsbruck, Innrain 52c, 6020 Innsbruck, Austria

## Abstract

A computational search for polymorphs of cytosine, 5-flucytosine and a 1 : 1 mixture of the two substances not only rationalised the preferred packing arrangements but also enabled the finding and characterisation of cytosine/5-flucytosine solid solutions. The structures of the new solid forms were determined by combining laboratory powder X-ray diffraction data and computational modelling.

The formation of multi-component systems, in particular co-crystals, has gained significant attention in crystal engineering fields, as their physicochemical properties can be altered and oftentimes enhanced.^1^,^2^ By adding an appropriate second component, it is possible to generate multi-component crystals of an active substance with improved solubility, bioavailability, hygroscopicity, physical and chemical stability, processability, *etc.* In contrast to salt formation, which requires ionisable components, the formation of solvates, co-crystals and solid solutions is introduced through a non-covalently bonded solvent molecule or neutral molecule (co-former), typically held together by hydrogen bonds.^3^

Solvent molecules or co-formers can occupy regular positions in the crystal lattice, leading to a well-defined stoichiometric ratio (*e.g.*, mono-, di- and hemi-) of the two or more components, best described as stoichiometric solvates (hydrates)^4^ or co-crystals, depending on the nature of the co-former.^5^ In contrast, a second substance (typically a structural homologue) may be incorporated into the crystal lattice substitutionally, by replacing the molecules of the first component in the lattice (isomorphism). Smaller molecules may also be incorporated interstitially by fitting into the space in the crystal lattice leading to a variable composition of the components, termed solid solution^6^ or nonstoichiometric solvate.^4^ Such non-stoichiometric behaviour, in particular of nonstoichiometric hydrates,^7^ complicates not only the processing, handling and storage of industrial materials but also the characterisation of the solid state forms.

Polymorphism screens aim at finding all (relevant) solid forms of a substance.^8^ Solution-based screening is the most common approach and routinely employed to identify accessible solid forms,^9^ although often applied on a trial and error basis to find the appropriate nucleation and growth conditions that lead to alternative forms. Crystallisation from the melt,^10^,^11^ sublimation,^12^ moisture sorption/desorption experiments,^13^ systematic desolvation (dehydration) studies^14^,^15^ and pressure crystallisation^16^–^18^ are other successful ways to obtain new forms. Furthermore, the use of seed crystals or additives to induce the formation of new forms by heterogeneous nucleation (templating, isomorphic seeding, *etc.*) has been shown to be very promising.^19^–^22^ The biggest problem with templating is the vast number of possibilities. Crystal structure prediction (CSP), which can be successfully implemented in (industrial) polymorphism screening programs,^15^,^23^–^26^ not only has the ability to warn us of putative alternative forms^27^,^28^ but may also help targeting these forms, *i.e.* selecting promising seeds/templates.^22^,^29^

In our recent study on hydrates of the two chemically related and pharmaceutically important compounds cytosine and 5-flucytosine (Fig. 1), we reported a monohydrate solid solution of these two substances. Preliminary dehydration studies of this monohydrate indicated the formation of two distinct solid forms with powder X-ray diffraction and IR spectroscopy data showing structural resemblance with the data of the two known 5-flucytosine anhydrates.^30^ This observation motivated us to take a closer look at the existence of isomorphic phases and isopolymorphs in this binary system. Isopolymorphism is a common phenomenon in structural homologues and describes phase systems, where each polymorph of one substance is isomorphous to a respective polymorphic form of another substance.^19^,^31^ Thus, such pairs of isomorphs may form mixed crystals (solid solutions).

Cytosine is known to be polymorphic (anhydrate **C-I:** Cambridge Structural Database^32^ ref-codes CYTSIN^33^ and CYTSIN01;^34^ **C-II**: CYTSIN02 (ref. 35)) and to form a monohydrate (ref-code family: CYTOSM^34^,^36^–^40^). Also, 5-flucytosine shows anhydrate polymorphism (**F-I**: MEBQEQ01 (ref. 41) and **F-II**: MEBQEQ^41^) and a rich solvate/hydrate crystal form landscape: hemihydrate,^42^ two monohydrates,^41^ hemipentahydrate^41^ and six solvates (methanol,^41^ ethanol,^30^ 2,2,2-trifluoroethanol,^41^ dimethyl acetamide,^42^ dimethyl sulfoxide^42^ and dimethyl formamide^30^).

The insolubility in most organic solvents as well as hydrate formation and decomposition at higher temperatures drastically limits the experimental space for polymorphism screens of each of the two compounds and also for co-crystals or solid solutions thereof. Our experimental screens^30^ (sections 2.3–2.5 of the ESI†) were limited to sublimation, slurry and dehydration studies and were therefore complemented with computational searches, CSP, for anhydrate (cytosine and 5-flucytosine) and 1 : 1 co-crystals. The crystal energy landscapes (*Z*′ = 1 & 2) for cytosine and 5-flucytosine, as well as the 1 : 1 cytosine/5-flucytosine crystal energy landscape (*Z*′ = 1) were generated by searches for the low energy minima of the keto tautomer on the lattice energy surface,^43^–^46^ and the final lattice energies were evaluated using DFT-D calculations.^47^–^51^ For more details, see section 1.1 of the ESI.†

The aim of this study was to unravel the possible molecular arrangements of neat cytosine, 5-flucytosine and mixed crystals thereof and to expand the solid form landscapes of these well-known compounds by combining experimental and computational polymorphism screening methods, extending previous studies.^30^,^41^

The structure of the known **C-I** anhydrate emerged as the global minimum structure on the computed crystal energy landscape (Fig. 2a and d), independent of the dispersion correction used. Based on experimental solid form screening and characterisation of the three cytosine solid forms, **C-I** was identified to be the thermodynamically most stable cytosine anhydrate at room temperature.^30^

The second cytosine polymorph, **C-II**, was found as a low energy structure on the crystal energy landscape, albeit +7.86 kJ mol^−1^ (rank 14) and +2.64 kJ mol^−1^ (rank 3) less stable than **C-I** using PBE-TS and PBE-D2, respectively. Analyses of the packing^52^ and hydrogen bonding motifs^53^ of the lowest-energy structures provide a unique insight into the possible and preferred packing arrangements. The majority of the lowest-energy cytosine structures forms the RM1-H ribbon motif (Fig. 2g), with adjacent ribbon motifs being linked through N–H⋯O hydrogen bonds to form three-dimensional (3D) hydrogen-bonded structures. An alternative hydrogen bonding motif (M2-H) could be identified among low energy structures, which can be related to RM1-H. In M2-H, one of the ${\text{R}}_{2}^{2}(8)$ dimers is replaced by ${\text{C}}_{2}^{2}(8)$ chains. Thus, RM-1H is the preferred building block for cytosine and is the common one-dimensional (1D) building block in the three experimental structures (**C-I**, **C-II** and monohydrate).

The 5-flucytosine anhydrate crystal energy landscape (Fig. 2b and e) has three structures which are more stable than the other computed packings, with the two experimental forms **F-I** and **F-II** being among those. Depending on the used dispersion correction, either **F-I** (D2) or **F-II** (TS) is indicated as the most stable polymorph at 0 K. Experimentally, the two polymorphs were found to be related enantiotropically,^54^ with **F-II** being the low temperature and room temperature form and 0.55 ± 0.02 kJ mol^−1^ more stable than **F-I**. The **F-II** to **F-I** phase transformation occurs at approx. 170 °C.^30^ PBE-TS (Fig. 2b) estimates the stability order correctly, **F-II** being 0.48 kJ mol^−1^ more stable than **F-I**, but has another packing as the global minimum structure (f3194), albeit only 0.24 kJ mol^−1^ more stable than **F-II**. The PBE-D2 energy landscape (Fig. 2e) has **F-I** as the global energy minimum and **F-II** as the rank 3 structure, 2.11 kJ mol^−1^ less stable, which may be the error of the applied method. Therefore, based on the calculations, it is not possible to conclude the stability order of the two forms. The f3194 structure was found to be the second most stable structure in Fig. 2e. The three lowest-energy structures share the same 1D supramolecular motif,^52^ the RM1-F chain (Fig. 2h). Exchanging the C5 hydrogen-atom (Fig. 1) with a fluorine-atom does not affect the preferentiality for the RM1 motif, but influences the 3D arrangement of the common building block (RM1-H and RM1-F). Alternative hydrogen bonding interactions of 5-flucytosine are possible, *e.g.* M3-F (Fig. 2h), but lead to less stable packing arrangements. The RM2 ribbon motif seen in 5-flucytosine monohydrate II (ref. 41) and other computed low energy monohydrate structures^30^ has not been observed among the low energy 5-flucytosine anhydrates, indicating that water is essential for its formation.

The cytosine and 5-flucytosine crystal energy landscapes exhibit candidate structures for alternative polymorphs, in particular c123 and f3194 (*.res files are given in the ESI†). The formation of the latter two may be complicated by the fact that the experimental structures have the same strong 1D packing arrangement and may convert to one of the experimental forms. Across the two compounds' crystal energy landscapes, isostructural packings can be identified. The cytosine crystal energy landscape has both experimental 5-flucytosine structures among the lowest-energy structures (**c1307** and **c304**), suggesting that isomorphic seeding or vapour deposition experiments^22^,^29^ may enable the formation of the two polymorphs. Similarly, the 5-flucytosine crystal energy landscape shows structures which correspond to the experimental cytosine polymorphs. Structure **f777** is in the energy range for a putative polymorph, but **f2266** is too high in energy to be expected as an observable form. Furthermore, the low energy structures c123 and f7, as well as f3194 and c4437, are isostructural.

Packing comparisons of the four experimental forms (Table 1), ignoring the C5 substitution, revealed that with the exception of the structure pair C-I/F-II, all possible pairs show only 1D similarity, the RM1 chain. **C-I** and **F-II** share 2D stacks of the RM1 motif.

The cytosine/5-flucytosine 1 : 1 “co-crystal” energy landscape (Fig. 2c and f) has the **F-I** and **F-II** packings, denoted as **CF-I** and C**F-II**, among the lowest-energy structures, but not the **C-I** and **C-II** packing arrangements among the low energy structures. All of the lowest-energy structures have the RM1 ribbon motif, either as a mixed type (RM1-HF, Fig. 2i) or a combination of RM1-H and RM1-F. The **CF-I** packing was found once in Fig. 2c and f, exhibiting the mixed ribbon motif, RM1-HF. In contrast, 13 computed structures in Fig. 2c and f are isostructural with **F-II**, differing in the relative location of the cytosine and 5-flucytosine molecules in the same packing arrangement. Furthermore, the structure cf21 is isostructural with c123 and f7 and cf3232 with f3194 and c4437. Based on the lattice energies, the three lowest energy packings, **CF-I**, **CF-II** and cf21, may be expected to be the experimental forms, either co-crystals or solid solutions.

Seeding experiments in methanol, acetonitrile and a THF/methanol mixture and vapour deposition experiments onto **C-I**, **C-II**, **F-I** and **F-II** crystals were attempted but unsuccessful in obtaining specific one-component isopolymorphs of cytosine or 5-flucytosine. This is not surprising for the high energy structure f2266, but we expected to get at least some indications for the existence of c304 or c1307. However, crystallisation experiments from mixed cytosine/5-flucytosine solutions resulted in a monohydrate solid solution from (water)^30^ and fine powder of **CF-I** and/or **CF-II** crystals (THF/methanol and acetonitrile). The PXRD (Fig. 3a) and IR data (Fig. 3b) of the latter two correspond to **CF-I** and **CF-II** phases obtained in systematic dehydration studies of the cytosine/5-flucytosine monohydrate solid solution (section 2.4 of the ESI†). The use of seed crystals allowed, to some extent, the control of the outcome of the crystallisation experiments.

Phase pure **CF-I** and **CF-II** samples were prepared from cytosine : 5-flucytosine starting ratios of 0.4 : 0.6. **CF-I** is obtained by slurring the two compounds in 1-butanol between 10 and 40 °C for two weeks. Slurrying the two compounds in water between 10 and 20 °C for three days led to the monohydrate.^30^ Dehydration of the monohydrate over P_2_O_5_ (0% RH) at temperatures ≤25 °C results in **CF-II**. Similar to the polymorphic pair **F-I**/**F-II**, the **CF-II** to **CF-I** phase transformation is observed as an endothermic peak in differential scanning calorimetry (DSC) experiments (Fig. 3c). The two forms (**CF-I** and **CF-II**) of the solid solution are enantiotropically related, with **CF-I** being the ambient and high temperature form and **CF-II** the low temperature form (section 2.3 of the ESI†). The transformation enthalpy of **CF-II** to **CF-I** was measured to be 0.7 ± 0.1 kJ mol^−1^ at 197.5 ± 6 °C (heating rate: 10 °C min^−1^).

The structures for **CF-I** and **CF-II** were determined using powder X-ray diffraction data (ESI,† Fig. S2) and computed structures to generate the starting geometry. The PBE-TS models were also used to refine the candidate structures. **CF-I** crystallises in the tetragonal space group *P*4_1_2_1_2 with one molecule in the asymmetric unit.‡ The cytosine : 5-flucytosine molar ratio was refined to 0.394(11) : 0.607(11). The structure has the RM1-HF ribbon motif (Fig. 2i), with ribbons stacking into columns (Fig. 4a). Adjacent columns are inclined by 67.5° and linked through N7–H⋯O hydrogen bonds to form a 3D hydrogen-bonding network structure. The **CF-I** packing is isostructural with **F-I** and corresponds to the third lowest energy packing in Fig. 2c and the lowest energy structure in Fig. 2f.

The second polymorph, **CF-II**, crystallises in the mono-clinic space group *P*2_1_/*n* with one molecule in the asymmetric unit.§ The structure exhibits the RM1 ribbon motif (Fig. 2i) with cytosine : 5-flucytosine occupancies of 0.359(9): 0.641(9). The ribbon motifs are linked through N7⋯H…O hydrogen bonds to adjacent ribbons (Fig. 4b), leading to sheets parallel to (102). As suggested from the PXRD and IR data, **CF-II** and **F-II** are isostructural and **CF-II** corresponds to the set of 13 structures in Fig. 2c and f, the second most stable packing using PBE-TS and the third most stable packing using PBE-D2. As seen for the 5-flucytosine polymorphs, the PBE-TS calculations correctly reproduce the experimental stability order (**CF-II** is slightly more stable than **CF-I**), whereas with PBE-D2, the 0 K stability order is inverted.

The question of whether a co-crystal or solid solution of cytosine and 5-flucytosine is present was addressed experimentally by using different molar ratios for preparing the mixed crystallisation products. Both forms, **CF-I** and **CF-II**, were obtained using different molar ratios of cytosine : 5-flucytosine, although approx. 20% of 5-flucytosine was required that the solid solutions emerged as stable forms, similar to that observed for the monohydrate solid solution.^30^ In addition to the two neat forms (**CF-I** and **CF-II**) and the monohydrate, the hemipentahydrate and several solvates were obtained (see the next paragraph). *Via* solvation/desolvation (water/solvent content determinations), it was confirmed that different molar ratios of cytosine and 5-flucytosine crystallised in the same packing arrangement (see also ref. 30), confirming the presence of solid solutions. The thermal decomposition did not allow us to determine the melting points of the non-solvated solid solutions. Therefore it was not possible to construct binary temperature/composition diagrams with different cytosine/5-flucytosine ratios. The computed crystal energy landscapes support the conclusion that **CF-I** and **CF-II** are solid solutions, as the packings can be found among the lowest-energy structures in all three crystal energy landscapes. Even though we were not able to produce the single component isopolymorphs of cytosine or 5-flucytosine, modelling, substitution calculations and CSP give us access to these structures. That cytosine can substitute the 5-flucytosine positions in **F-I** and **F-II** can be related to the fact that both structures are the lowest-energy structures in Fig. 2b and e (5-flucytosine) and Fig. 2c and f (cytosine/5-flucytosine). Also, the fact that more than one structure on Fig. 2c and f corresponds to the **F-II** packing indicates that a solid solution is formed.

The limited experimental solvent screen for cytosine/5-flucytosine solid solutions resulted, in addition to the novel anhydrates, in the monohydrate I, the hemipentahydrate and four solvate forms: two hemisolvates with methanol and ethanol and two monosolvates with dimethyl sulfoxide and dimethyl formamide (section 2.7 of the ESI†). The methanol solvate exhibits low stability and desolvates on storage under ambient conditions within one day, while the ethanol, DMSO and DMF solvates remain unchanged for approx. five to seven days. Based on the fact that the four cytosine/5-flucytosine solvates are isostructural with the corresponding 5-flucytosine solvates, the existence of two other solvates (2,2,2-trifluoroethanol and dimethyl acetamide) and two additional hydrates (hemihydrate and monohydrate II) can be predicted with certainty.

## Conclusions

**Fig. 5:**
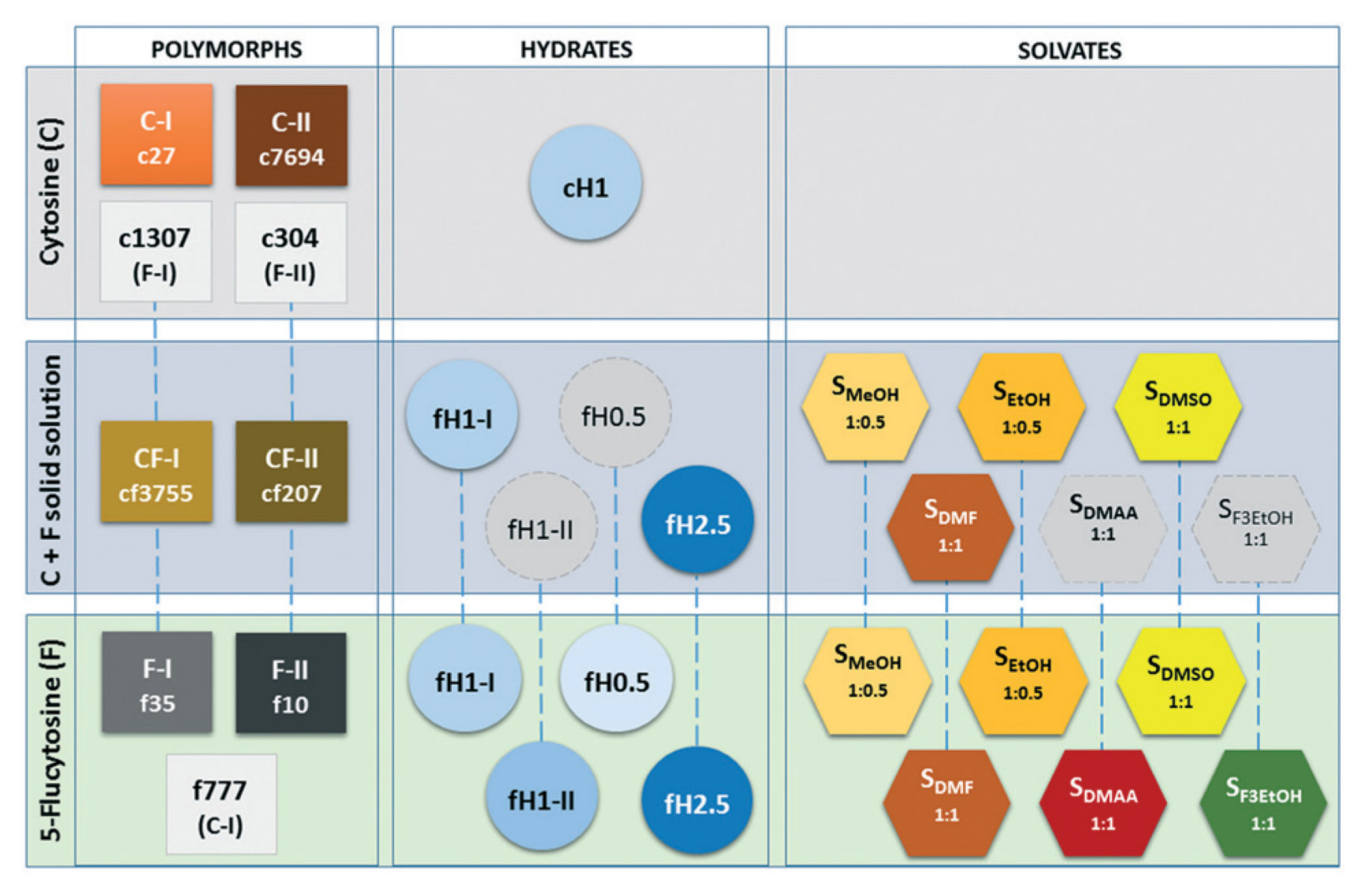
Overview over cytosine (C), 5-flucytosine (F) and mixed solid forms. Isopolymorphs, with the exception of C-I and f777, are connected with blue dashed lines. Structures c1307, c304 and f777 are isopolymorphs that have not been observed yet, but are feasible kinetic forms. Grey symbols indicate the hydrates and solvates of the solid solution that are likely to exist as well.

For all solid forms of 5-flucytosine which we attempted to co-crystallise with cytosine, the molecule can be substituted with cytosine, as experimentally shown for **F-I**, **F-II**, hemipenta- and monohydrate I (ref. 30) and four solvates, leading to solid solutions (Fig. 5). Thus, we can assume that the same holds for the remaining four 5-flucytosine forms. The presence of 5-flucytosine is essential for the formation of the 3D packings. Computationally, the formation of solid solutions could be rationalised based on the fact that the experimental structures were found in the cytosine, 5-flucytosine and mixed cytosine/5-flucytosine crystal energy landscapes and that the anhydrate solid solutions (**CF-I** and **CF-II**) and 5-flucytosine anhydrates (**F-I** and **F-II**) are the lowest-energy structures.

The computed crystal energy landscapes doubtlessly suggest that other anhydrate polymorphs exist. It is very likely that the putative neat forms exhibit the RM1 ribbon motif. The hydrogen ↔ fluorine atom exchange in the anhydrous compounds does not affect the predominance of the RM1 ribbon motif. The most promising candidate for another 5-flucytosine polymorph, different from a cytosine isopolymorph, is the structure f3194 and for another solid solution cf21, with 5-flucytosine molecules replacing cytosine positions (structure c123).

To conclude, this study is another successful demonstration of the complementarity of computational and experimental screening and characterisation methods, allowing targeting crystallisation using isomorphous seeds/templates to produce novel solid forms and supporting structure solution from laboratory powder X-ray diffraction data.

## Supplementary Material

† Electronic supplementary information (ESI) available: Computational generation of the anhydrate crystal energy landscapes; preparation and solvent screen of anhydrate solid solutions; Rietveld refinement; solvates of cytosine/5-flucytosine. See DOI: 10.1039/c7ce00939a

ESI

## Figures and Tables

**Fig. 1:**
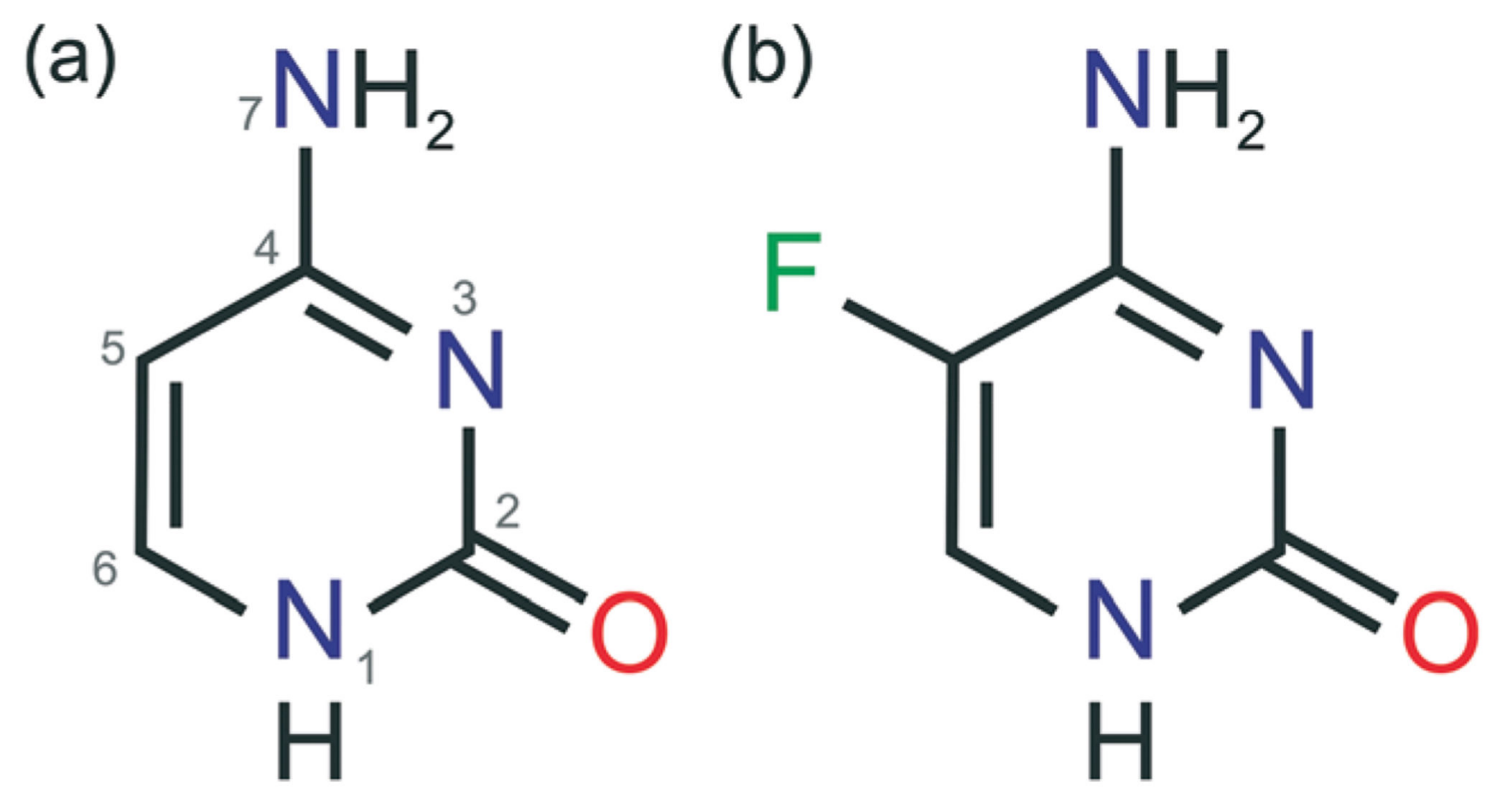
Molecular diagrams of (a) cytosine and (b) 5-flucytosine. The atom numbering system given in (a) is used consistently throughout this work.

**Fig. 2:**
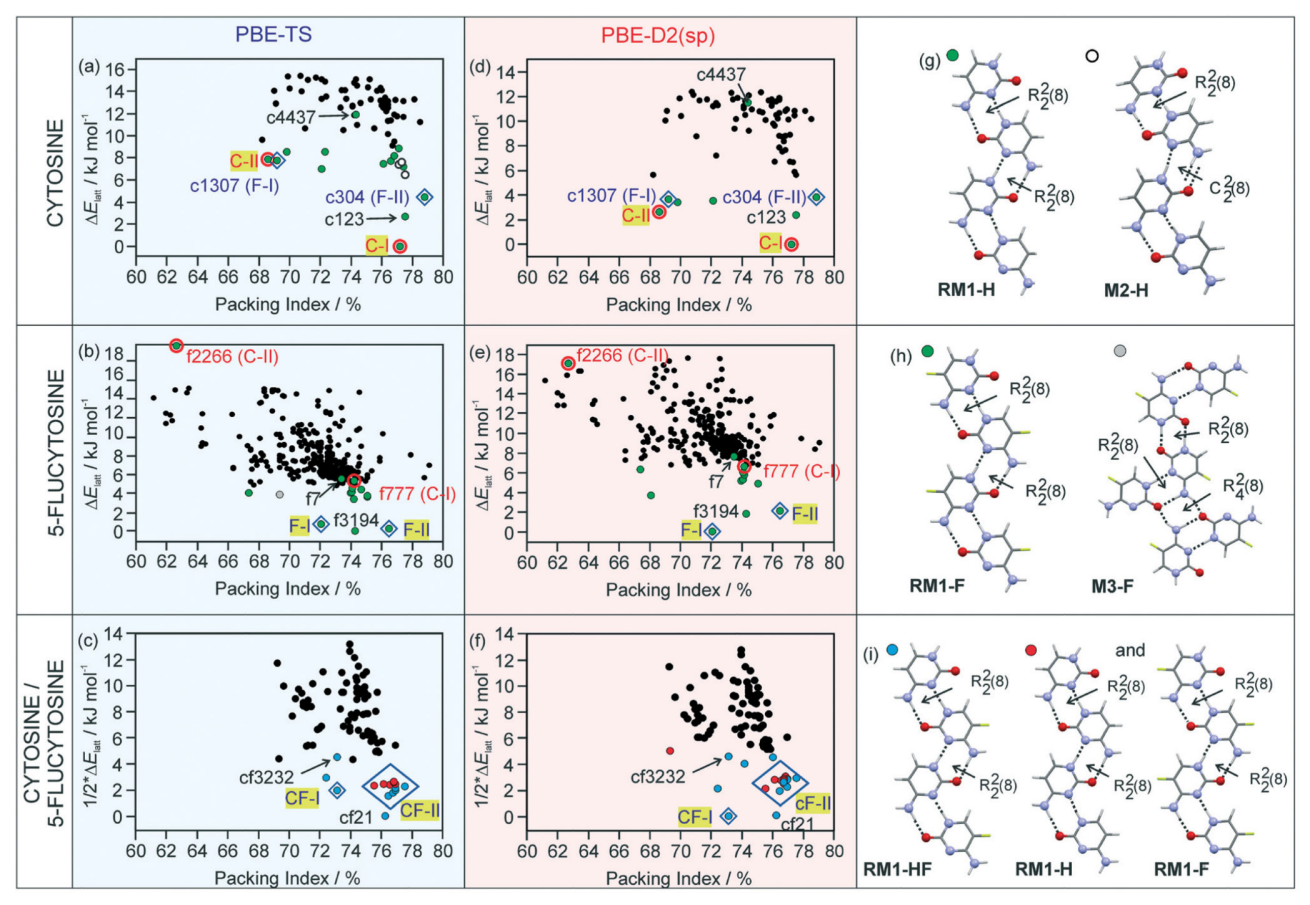
Computed crystal energy landscapes for (a and d) cytosine, (b and e) 5-flucytosine and (c and f) a 1 : 1 mixture of cytosine and 5-flucytosine. In (a–c) the PBE-TS lattice energies are given and in (d–f) the single point PBE-D2 lattice energy estimations of the PBE-TS structures. Each symbol denotes a crystal structure. The experimental structures are encircled or marked with a diamond and the labels highlighted in yellow (**C-I**, **C-II**, **F-I**, **F-II**, **CF-I**, and **CF-II**). Computed structures that are isostructural with the experimental cytosine and 5-flucytosine packings are encircled or marked with a diamond and labelled as follows: c1307 (isostructural with **F-I**), c304 (**F-II**), f777 (**C-**I) and f2266 (**C-II**), with c or f corresponding to cytosine or 5-flucytosine, respectively, and the number to the initial rank (CrystalPredictor). In (g–i), the hydrogen-bonding motifs found among the lowest-energy structures are shown.

**Fig. 3:**
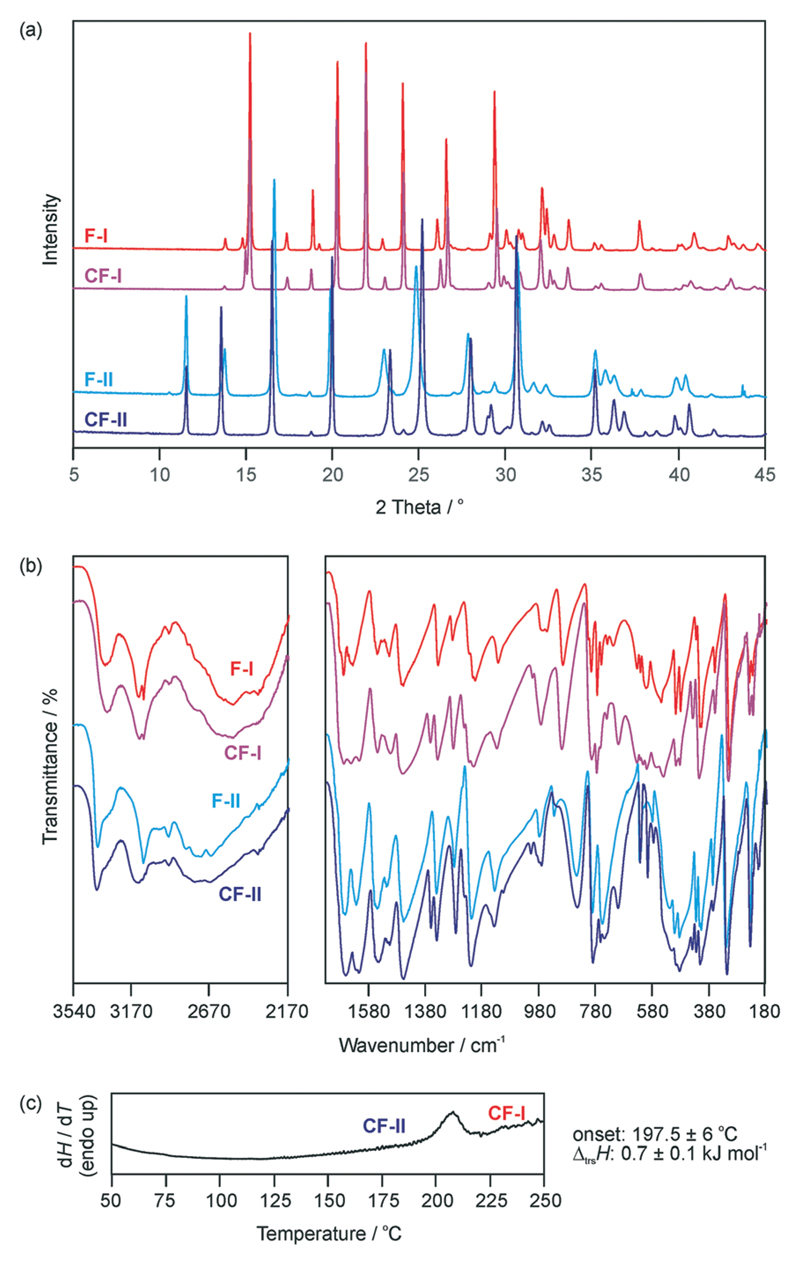
(a) Powder X-ray diffractograms and (b) IR spectra of 5-flucytosine and cytosine/5-flucytosine anhydrates. (c) DSC curve showing the cytosine/5-flucytosine anhydrate II (**CF-II**) to anhydrate I (**CF-I**) transformation.

**Fig. 4:**
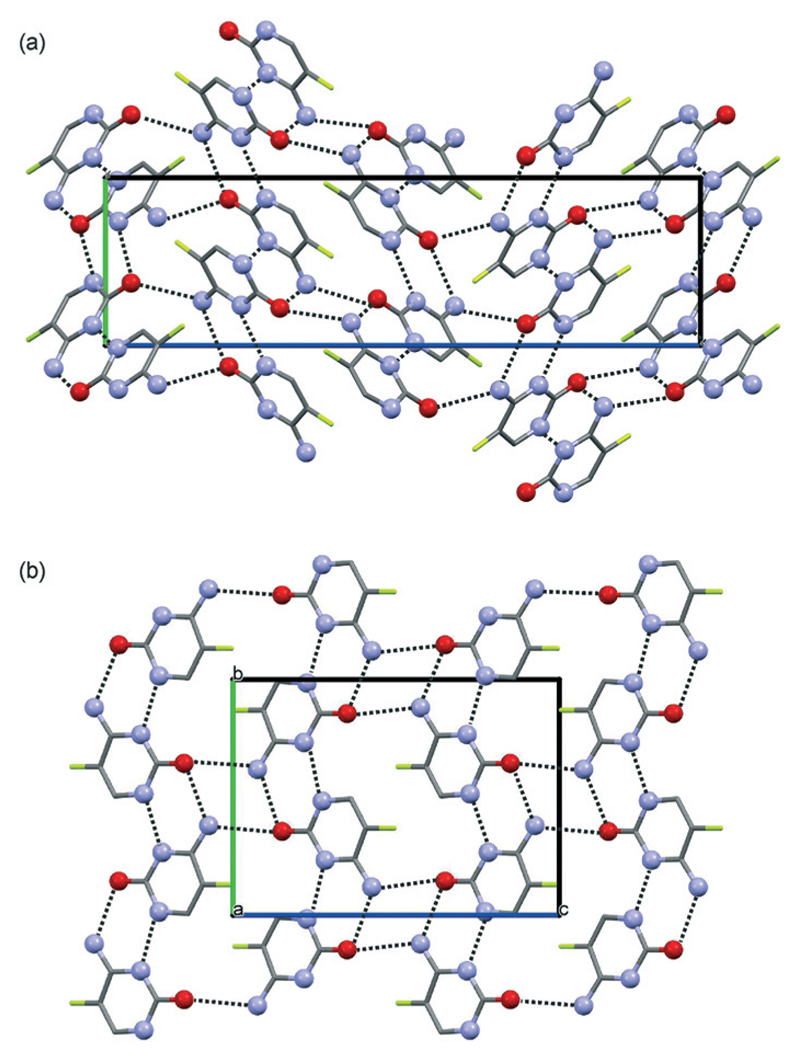
Crystal packing of (a) cytosine/5-flucytosine anhydrate I and (b) cytosine/5-flucytosine anhydrate II. The two packing diagrams are viewed along the *a* crystallographic axes. Hydrogen-bonding is indicated by dotted lines. Hydrogen atoms are omitted for clarity. Fluorine atom positions are not fully occupied.

**Table 1:** Quantification^a^ of the similarity of cytosine and 5-flucytosine crystal structures (PBE-TS) showing the packing similarity (rmsd_*n*_)^55^ and common packing motif (RM1)

	C-I	C-II	F-I	F-II
**C-I**	—	5(0.316)	3(0.197)	9(0.211)
**C-II**	RM1	—	5(0.189)	5(0.288)
**F-I**	RM1	RM1	—	5(0.197)
**F-II**	RM1 stacks	RM1	RM1	—

a The bold numbers indicate the number of molecules, *n*, that match within the distance and angle tolerances of 20% and 20°, respectively, with the rmsd_*n*_ values in brackets. RM1 – ribbon motif (Fig. 2g and i).

## Footnotes

‡ Crystal data of **CF-I**: C_4_H_4.393_F_0.607_N_3_O, Mr = 122.023, tetragonal, *P*4_1_2_1_2, *T* = 25 °C, sample formulation: powder, wavelength: 1.54184 Å, *a* = 6.67372(5) Å, *c* = 23.6290(3) Å, *Z* = 8, density = 1.540 g cm^−3^, 2 theta range for data collection: 2 to 70°, background treatment: Chebyshev polynomial, no. of measured reflections: 135, refinement method: Rietveld, data/parameter/restraints: 135/74/40, goodness of fit: 1.98 (on *Y*_obs_), *R*_wp_ = 6.29%, *R*_exp_ = 3.17%, *R*_p_ = 4.45%.

§ Crystal data of **CF-II**: C_4_H_4.359_F_0.641_N_3_O, Mr = 122.635, monoclinic, *P*2_1_/*n*, *T* = 25 °C, sample formulation: powder, wavelength: 1.54184 Å, *a* = 7.00808(9) Å, *b* = 9.43931(11) Å, *c* = 13.0306(2) Å, *β* = 91.179(2)°, *Z* = 4, density = 1.653 g cm^−3^, 2 theta range for data collection: 2 to 70°, background treatment: Chebyshev polynomial, no. of measured reflections: 163, refinement method: Rietveld, data/parameter/restraints: 163/69/40, goodness of fit: 1.80 (on *Y*_obs_), *R*_wp_ = 6.05%, *R*_exp_ = 3.36%, *R*p = 4.41%.
