# Supplementary material for: Prediction and experimental validation of solid solutions and isopolymorphs of cytosine/5-flucytosine†
Source: CrystEngComm. Author manuscript; Available in PMC 2018 Nov 5. (PMC6218006; doi:10.1039/C7CE00939A)
Supplement: ESI [file NIHMS80018-supplement-ESI.pdf]

Prediction and Experimental Validation of Solid Solutions and Isopolymorphs of Cytosine/5-Flucytosine

Doris E. Braun & Ulrich J. Griesser

Electronic Supplementary Information

Contents

1. COMPUTATIONAL 2
1.1. Computational Generation of the Anhydrate Crystal Energy Landscapes 2
1.1.1. Cytosine and 5-Flucytosine Tautomer Selection 2
1.1.2. Structure Generation: CrystalPredictor 2
1.1.3. Reminimisation: DMACRYS 2
1.1.4. Reminimisation: CrystalOptimizer 2
1.1.5. Reminimisation: CASTEP (PBE-TS and PBE-D2) 3
1.2. Computationally Generated Low-Energy Structures 5
1.2.1. Cytosine Low-Energy Structures 5
1.2.2. 5-Flucytosine Low-Energy Structures 7
1.2.3. Cytosine/5-Flucytosine Low-Energy Structures 11
1.3. Representation of the Experimental Structures 13
1.3.1. Cytosine 13
1.3.2. 5-Flucytosine 13
1.4. Selected Computed Low-Energy Structures (Possible Polymorphs) 14
1.4.1. Cytosine: c123 14
1.4.2. 5-Flucytosine: f3194 15
1.4.3. Cytosine/5-Flucytosine: ss21 16
2. EXPERIMENTAL 17
2.1. Preparation of Anhydrate Solid Solutions 17
2.2. Methodology 17
2.2.1. Thermal Analysis 17
2.2.2. Infrared Spectroscopy 18
2.2.3. Powder X-ray Diffraction 18
2.3. Solvent Screen 18
2.3.1. Crystal16® Cycling Experiments 18
2.3.2. Slurry Experiments 19
2.4. Dehydration Experiments 20
2.5. Sublimation Experiments 20
2.6. Structure Determination: Simulated Annealing and Rietveld Refinement 21
2.6.1. Solid Solution Anhydrate I (ssF-I) 21
2.6.2. Solid Solution Anhydrate II (ssF-II) 22
2.7. Solvates 26
2.7.1. Methanol Solvate 26
2.7.2. Ethanol Solvate 27
2.7.3. Dimethyl Formamide Solvate 29
2.7.4. Dimethyl Sulfoxide Solvate 30
2.8. PXRD Comparisons: 5-Flucytosine and Cytosine/5-Flucytosine Solid Solutions 32
3. OVERVIEW SOLID FORMS 34

1. COMPUTATIONAL

1.1. Computational Generation of the Anhydrate Crystal Energy Landscapes

1.1.1. Cytosine and 5-Flucytosine Tautomer Selection

The two investigated compounds are known to exist (at least in solution) in different tautomeric forms. A survey of the two compounds' structures present in the Cambridge Structural Database[1] revealed that so far only the keto (amino-keto) tautomer has been identified in solid state. Thus, only the keto tautomer was considered in our computational searches for anhydrate polymorphs.

1.1.2. Structure Generation: CrystalPredictor

Cytosine (Z' = 1 & 2), 5-flucytosine (Z' = 1 & 2), and cytosine/5-flucytosine (1:1, Z' =1) crystal structures were generated with the program CrystalPredictor.[2-4] For *cyt* and *fcyt* each 650.000 structures (150.000 Z' = 1 and 500.000 Z' = 2) and for cytosine/5-flucytosine 500.000 structures were randomly generated in following 48 space groups, keeping the molecular geometry rigid (keto tautomer): $P1$, $P\bar{1}$, $P2_1$, $P2_1/c$, $P2_12_12$, $P2_12_12_1$, $Pna2_1$, $Pca2_1$, $Pbca$, $Pbcn$, $C2/c$, Cc, $C2$, Pc, Cm, $P2_1/m$, $C2/m$, $P2/c$, $C222_1$, $Pmn2_1$, $Fdd2$, $Pnna$, $Pccn$, $Pbcm$, $Pnnm$, $Pmmn$, $Pnma$, $P4_1$, $P4_3$, $I\bar{4}$, $P4/n$, $P4_2/n$, $I4/m$, $I41/a$, $P41212$, $P4_32_12$, $P3_1$, $P3_2$, $R3$, $P\bar{3}$, $R\bar{3}$, $P3_121$, $P322_1$, $R3c$, $R\bar{3}c$, $P6_1$, $P6_3$, $P6_3/m$.

The structures were relaxed to a local minimum in intermolecular lattice energy, calculated from the FIT[5] exp-6 repulsion-dispersion potential and atomic charges, fitted to electrostatic potential around the PBE0/aug-cc-pVTz charge density using the CHELPG scheme.[6]

1.1.3. Reminimisation: DMACRYS

For each of the searches the lowest energy structures (Table S1) were refined using DMACRYS[7] with a more realistic, distributed multipole model[8] for the electrostatic forces which had been derived using GDMA2[9] to analyse the PBE0/aug-cc-pVTz charge density.

1.1.4. Reminimisation: CrystalOptimizer

The orientation of the amino group (planar vs. pyramidal orientation) of the most stable structures (Table S1) of the two compounds was optimised with the program CrystalOptimizer.[10] Conformational energy penalties and isolated molecule charge densities were computed at the PBE0/aug-cc-pVTz level of theory.

Table S1. Overview Computational Generation of the Anhydrate Crystal Energy Landscapes.

	**Cytosine**	**5-Flucytosine**	**Cytosine/5-Flucytosine**
CrystalPredictor (rigid body)			
Charges	PBE0/aug-cc-pVTz	PBE0/aug-cc-pVTz	PBE0/aug-cc-pVTz
Structures	150000 (Z'=1)<br>500000 (Z'=2)	150000 (Z'=1)<br>500000 (Z'=2)	500000 (Z'=1, Z''=2)
DMACRYS (rigid body)			
Multipoles	PBE0/aug-cc-pVTz	PBE0/aug-cc-pVTz	PBE0/aug-cc-pVTz
Energy range	20.0 kJ mol^{-1} (Z'=1)<br>10.1 kJ mol^{-1} (Z'=2)	20.0 kJ mol^{-1} (Z'=1)<br>12.0 kJ mol^{-1} (Z'=2)	10.7 kJ mol^{-1}
Structures	3928 (Z'=1)<br>10000 (Z'=2)	3153 (Z'=1)<br>5272 (Z'=2)	10000
CrystalOptimizer (flexible)			
Multipoles	PBE0/aug-cc-pVTz	PBE0/aug-cc-pVTz	PBE0/aug-cc-pVTz
Energy range	20.0 kJ mol^{-1} (Z'=1)<br>16.0 kJ mol^{-1} (Z'=2)	15.0 kJ mol^{-1} (Z'=1)<br>12.0 kJ mol^{-1} (Z'=2)	15.0 kJ mol^{-1}
Structures	219 (Z'=1)<br>658 (Z'=2)	573 (Z'=1)<br>2354 (Z'=2)	1345
CASTEP PBE-TS (cut-off: 780 eV, k-points: 0.07)			
Energy range	15.0 kJ mol^{-1} (Z'=1)<br>15.0 kJ mol^{-1} (Z'=2)	11.0 kJ mol^{-1} (Z'=1)<br>**5.0** kJ mol^{-1} (Z'=2)	11.0 kJ mol^{-1}
Structures	38 (Z'=1)<br>138 (Z'=2)	155 (Z'=1)<br>172 (Z'=2)	90
CASTEP PBE-D2 (cut-off: 780 eV, k-points: 0.07, single point calculations)			
Energy range	15.0 kJ mol^{-1} (Z'=1)<br>15.0 kJ mol^{-1} (Z'=2)	15.0 kJ mol^{-1} (Z'=1)<br>**all** (Z'=2)	all
Structures	18 (Z'=1)<br>77 (Z'=2)	125 (Z'=1)<br>172 (Z'=2)	90

1.1.5. Reminimisation: CASTEP (PBE-TS and PBE-D2)

The DFT-D calculations were carried out with the CASTEP plane wave code[11] using the Perdew-Burke-Ernzerhof (PBE) generalised gradient approximation (GGA) exchange-correlation density functional[12] and ultrasoft pseudopotentials,[13] with the addition of a semi-empirical dispersion correction, either the Tkatchenko and Scheffler (TS) model,[14] or Grimme06 (D2).[15] In a first step, the structures were geometry optimised using the TS dispersion correction. Brillouin zone integrations were performed on a symmetrised Monkhorst–Pack *k*-point grid with the number of *k*-points chosen to provide a maximum spacing of 0.07 Å^{-1} and a basis set cut-off of 780 eV. The self-consistent field convergence on total energy was set to 1x10^{-5} eV. Energy minimisations were performed using the Broyden–

Fletcher–Goldfarb–Shanno optimisation scheme within the space group constraints. The optimisations were considered complete when energies were converged to better than $2x10^{-5}$ eV per atom, atomic displacements converged to $1x10^{-3}$ Å, maximum forces to $5x10^{-2}$ eV Å^{-1}, and maximum stresses were converged to $1x10^{-1}$ GPa. The energies for the structures were recalculated, without optimisation, with the number of *k*-points chosen to provide a maximum spacing of 0.07 Å^{-1} and a basis set cut-off of 780 eV, using the D2 dispersion correction. Isolated molecule minimisations to compute the isolated cytosine (keto tautomer) and 5-flucytosine (keto tautomer, U_{gas}) were performed by placing a single molecule in a fixed cubic 35x35x35 Å^3 unit cell, then optimised with the same settings as used for the crystal calculations.

H ↔ F exchange was systematically applied to the experimental structures **C-I**, **C-II, F-I** and **F-II** to produce isostructural cytosine, 5-flucyosine and mixed crystal structures thereof. The structures were minimised as described above.

1.2. Computationally Generated Low-Energy Structures

All calculated structures are available in .res format from the authors on request.

1.2.1. Cytosine Low-Energy Structures

Table S2. Hypothetical Low-Energy Crystal Structures of Cytosine Anhydrates (PBE-TS and PBE-D2 energies). Experimental structures are highlighted in green.

ID[a]	Space Group	a	b	c	α	β	γ	PI[b]	E_{latt}	ΔE_{latt}
		/ Å			/ °				/ kJ mol^{-1}	
c27 (**C-I**)	$P2_12_12_1$	3.783	9.486	12.846	90	90	90	77.2	-169.84	0.00
c123	$P2_1/c$	9.418	3.729	26.260	90	90.29	90	77.5	-167.45	2.38
c7694 (**C-II**)	*Pccn*	14.949	14.990	9.292	90	90	90	68.6	-167.20	2.64
c825	$P\text{-}42_1c$	10.429	10.429	9.397	90	90	90	69.8	-166.42	3.42
c5882	$P2_12_12_1$	5.361	7.787	23.643	90	90	90	72.1	-166.27	3.56
c1307 (**cF-I**)	$P4_32_12$	6.677	6.677	23.041	90	90	90	69.20	-166.17	3.66
c304 (**cF-II**)	$P2_1/n$	3.681	9.369	13.263	90	94.81	90	78.8	-166.02	3.82
c1245 (**cF-I**)	$P4_12_12$	6.721	6.721	22.867	90	90	90	68.8	-165.96	3.87
c6792	$P2_12_12$	9.486	11.772	9.371	90	90	90	68.2	-164.20	5.63
c1062	$Pna2_1$	18.239	3.754	13.469	90	90	90	77.5	-164.18	5.65
c65	$Pna2_1$	27.538	3.664	9.174	90	90	90	77.4	-163.95	5.89
c9095	$P2_1/n$	15.710	3.802	17.192	90	115.31	90	77.3	-163.18	6.66
c2083	$Pca2_1$	26.814	3.645	9.567	90	90	90	76.1	-163.12	6.71
c1130	$P2_1/c$	14.020	3.759	17.733	90	94.71	90	77.1	-163.10	6.74
c52	$P2_12_12_1$	4.097	9.334	12.830	90	90	90	72.3	-162.63	7.20
c312	$P2_1/n$	3.707	26.973	9.361	90	90.73	90	76.6	-162.31	7.53
c277	$P2_1/n$	3.732	26.817	9.343	90	91.49	90	76.8	-162.16	7.68
c868	$P2_1/c$	7.487	9.587	7.246	90	115.43	90	76.4	-161.98	7.86
c410	$Pna2_1$	19.038	3.649	13.418	90	90	90	76.7	-161.47	8.36
c2606	*Pbca*	9.702	7.051	27.378	90	90	90	76.6	-161.10	8.73
c2980	$P2_1/c$	8.251	15.933	8.189	90	111.11	90	71	-161.07	8.77
c4535	$P2_1/n$	16.282	3.638	16.749	90	109.95	90	76.8	-161.03	8.81
c1400	$P2_1/n$	3.709	13.917	9.098	90	91.40	90	76.4	-161.02	8.82
c121	$P2_1/c$	3.684	9.311	27.233	90	93.61	90	77.1	-160.94	8.90
c6962	$P2_1$	8.818	3.787	14.549	90	99.36	90	74.4	-160.61	9.23
c235	$Pna2_1$	26.560	9.350	3.907	90	90	90	73.5	-160.44	9.39
c4688	Pbca	13.878	9.738	13.942	90	90	90	76.1	-160.21	9.63
c4024	Pbca	9.737	7.013	13.800	90	90	90	76	-160.02	9.82
c126	$P2_1/c$	9.364	11.607	10.063	90	108.44	90	69	-159.79	10.04
c260	$Pna2_1$	13.552	3.732	9.381	90	90	90	75.5	-159.74	10.10
c1867	*C2/c*	27.625	3.706	18.268	90	95.25	90	77	-159.70	10.13
c6612	$Pna2_1$	26.676	3.639	9.428	90	90	90	78.5	-159.65	10.19
c185	$P2_1/n$	10.137	9.387	10.356	90	96.89	90	73.6	-159.64	10.19
c9043	$P2_1/c$	3.707	8.889	28.562	90	91.72	90	76.3	-159.61	10.23
c9733	$P2_1/c$	9.287	9.730	11.549	90	100.46	90	70	-159.40	10.43
c454	*I2/a*	18.243	3.665	27.812	90	94.49	90	77.4	-159.35	10.49
c987	*Pbca*	9.780	6.963	13.829	90	90	90	76.2	-159.32	10.52
c5280	*C2/c*	18.545	3.766	28.000	90	100.16	90	74.2	-159.31	10.52
c7982	*P*-1	7.127	7.775	9.019	85.75	70.98	84.95	76.4	-159.15	10.68

ID[a]	Space Group	a	b	c	α	β	γ	PI[b]	E_{latt}	ΔE_{latt}
		/ Å			/ °				/ kJ mol^{-1}	
c1010	$Pna2_1$	9.727	13.438	3.625	90	90	90	75.3	-159.12	10.71
c409	*Pbca*	14.002	7.133	19.156	90	90	90	75.1	-159.03	10.81
c1371	*C*2/*c*	18.738	3.694	27.021	90	94.82	90	77.4	-159.01	10.83
c1616	*C*2/*c*	22.793	5.455	16.999	90	101.61	90	69.1	-159.01	10.83
c408	*Fdd*2	15.694	30.103	3.897	90	90	90	78.1	-159.00	10.83
c1077	*Pc*	7.074	3.800	17.595	90	97.68	90	76.8	-158.99	10.85
c1577	$P2_1/n$	8.794	3.777	29.192	90	98.60	90	74.6	-158.86	10.98
c79	$Pca2_1$	18.909	3.631	13.612	90	90	90	76.9	-158.85	10.98
c3087	$P2_1/c$	14.343	9.365	7.431	90	104.95	90	74.1	-158.80	11.04
c9031	*P*-1	6.675	7.191	11.499	96.99	92.09	114.75	72.1	-158.62	11.22
c440	$Pca2_1$	13.484	9.395	7.918	90	90	90	72.1	-158.61	11.22
c7843	$Pca2_1$	7.494	9.368	13.737	90	90	90	74.1	-158.60	11.23
c3382	$P2_1/n$	7.515	9.427	13.499	90	98.19	90	75.9	-158.58	11.26
c3433	$P2_1/c$	8.004	15.735	8.372	90	110.44	90	72.4	-158.55	11.28
c5526	$P2_1/n$	9.363	11.232	9.956	90	108.32	90	72.3	-158.37	11.47
c1821	$P2_1/n$	9.551	7.585	14.018	90	107.54	90	74.3	-158.33	11.50
c4437	*C*2/*c*	17.476	6.928	16.103	90	98.25	90	74.3	-158.33	11.51
c8089	$P2_1/c$	14.472	9.539	7.054	90	103.72	90	76	-158.29	11.55
c581	*P*2/*c*	9.411	3.686	27.271	90	90.31	90	76.2	-158.24	11.60
c5158	$P2_1/c$	9.526	7.799	13.056	90	93.55	90	74.2	-158.21	11.62
c7281	$P2_1/n$	9.484	7.850	13.101	90	103.30	90	75.7	-158.18	11.65
c8085	$P2_1/n$	10.381	9.271	10.742	90	101.95	90	71.2	-158.15	11.69
c84	$Pca2_1$	18.960	3.607	13.721	90	90	90	76.4	-158.13	11.70
c924	$P2_1$	6.910	7.903	9.305	90	99.91	90	71.7	-158.10	11.73
c8866	$P2_1/c$	9.551	12.815	7.745	90	90.95	90	75.9	-158.09	11.75
c2593	*C*2/*c*	18.544	3.803	27.710	90	93.19	90	73.4	-158.06	11.77
c9209	$P2_12_12_1$	9.206	9.312	11.941	90	90	90	69.9	-158.00	11.84
c8506	$P2_1/n$	16.429	3.619	16.890	90	110.63	90	76.4	-157.98	11.86
c7726	$P2_1/n$	4.081	9.343	25.389	90	90.76	90	73.7	-157.97	11.86
c3303	*P*-1	6.640	7.676	9.531	91.04	94.14	94.72	74.4	-157.76	12.07
c1928	*I*2/*a*	16.084	3.941	17.330	90	113.41	90	70.8	-157.73	12.11
c8067	$P2_12_12_1$	3.827	9.324	27.380	90	90	90	73.3	-157.53	12.30
c3483	*Pbca*	9.650	13.716	14.418	90	90	90	75.2	-157.52	12.31
c7455	$P2_1/c$	9.406	9.750	11.072	90	91.13	90	70.7	-157.48	12.36
c1588 (**dehy**)	$P2_1/c$	7.343	9.469	7.024	90	95.81	90	74.1	-154.45	15.38

[a]Structure ID: c – cytosine and rank CrystalPredictor. The CASTEP minimised structures were checked for higher symmetry using PLATON.[16] **cF-I** and **cF-II** – isostructural with **F-I** and **F-II**; **dehy** – isostructural with cytosine monohydrate. [b]Packing Index (%) calculated using PLATON.

1.2.2. 5-Flucytosine Low-Energy Structures

Table S3. Hypothetical Low-Energy Crystal Structures of 5-Flucytosine Anhydrates (PBE-TS and PBE-D2 energies). Experimental structures are highlighted in green.

ID[a]	Space Group	a	b	c	α	β	γ	PI[b]	E_{latt}	ΔE_{latt}
		/ Å			/ °				/ kJ mol^{-1}	
f35 (**F-I**)	$P4_12_12$	6.688	6.688	23.354	90	90	90	72.1	-144.10	0.00
f3194	$C2/c$	17.461	6.933	17.091	90	101.69	90	74.3	-142.29	1.81
f10 (**F-II**)	$P2_1/c$	4.035	9.465	12.912	90	90.36	90	76.5	-141.99	2.11
f1558	$Pbca$	9.340	9.578	24.718	90	90.00	90	68.1	-140.46	3.64
f337	$P2_1/c$	9.059	9.415	12.076	90	101.70	90	75.1	-139.27	4.84
f947	$P2_12_12_1$	5.105	7.739	25.943	90	90	90	74	-138.92	5.18
f3279	$P2_1$	8.018	4.599	14.332	90	105.15	90	74.1	-138.85	5.26
f2508	P-1	6.956	8.872	9.011	80.28	70.16	78.31	74.2	-138.33	5.77
f971	$C2/c$	13.455	9.397	17.225	90	110.22	90	74.1	-138.07	6.03
f293	$P2_1/c$	17.309	3.634	18.164	90	102.17	90	67.4	-137.82	6.28
f777 (**fC-I**)	$P2_12_12_1$	4.188	9.400	12.876	90	90	90	74.2	-137.50	6.60
f577	$Fdd2$	21.015	9.999	9.615	90	90	90	74.3	-137.38	6.73
f533	$C2/c$	9.238	9.361	24.752	90	90.53	90	70.6	-137.35	6.75
f150	$C2/c$	26.374	3.637	22.663	90	94.07	90	69.4	-137.35	6.76
f369	$P2_1$	8.378	3.716	16.228	90	96.34	90	75.1	-137.31	6.79
f1121	$Pbca$	9.275	9.198	25.108	90	90	90	70.5	-137.19	6.91
f1332	$C2/c$	12.019	10.037	17.787	90	100.77	90	71.6	-137.18	6.92
f29	$P2_1/c$	4.160	9.324	26.122	90	90.99	90	74.7	-137.14	6.96
f692	$P2_1/c$	12.544	9.333	9.165	90	90.03	90	70.4	-137.04	7.06
f537	$C2$	17.915	3.979	16.390	90	117.06	90	72.5	-136.97	7.14
f5313	$C2/c$	18.337	3.793	29.483	90	98.46	90	74.3	-136.92	7.19
f1088	$Pccn$	9.380	24.647	9.248	90	90	90	70.6	-136.90	7.21
f480	$C2/c$	17.640	6.993	18.424	90	94.41	90	66.4	-136.82	7.28
f86	P-1	4.178	9.328	14.083	105.32	90.33	93.35	71.3	-136.80	7.30
f679	$Pbca$	8.930	9.299	12.386	90	90	90	73.9	-136.69	7.41
f164	$P2_1/n$	16.315	3.736	18.304	90	93.15	90	67.5	-136.68	7.43
f93	$P2_1/c$	9.163	12.050	9.803	90	97.98	90	70.3	-136.60	7.50
f59	$P2_1/c$	4.077	27.004	9.332	90	91.48	90	73.7	-136.59	7.52
f127	$Pbca$	12.247	9.347	17.706	90	90	90	74.7	-136.52	7.59
f1036	P-1	7.045	8.826	9.234	79.72	76.81	68.64	73.1	-136.51	7.59
f371	Cc	6.233	9.034	9.274	90	92.33	90	72.8	-136.46	7.64
f125	$P2_1/c$	13.751	3.627	22.286	90	92.60	90	67.7	-136.40	7.70
f580	$P2_1/c$	9.275	8.965	12.797	90	104.24	90	73.5	-136.40	7.71
f7	$P2_1/c$	9.268	3.827	29.188	90	95.52	90	73.5	-136.39	7.72
f563	$P2_1/c$	12.791	8.940	9.301	90	104.89	90	73.7	-136.37	7.74
f19	$P2_12_12_1$	3.944	9.154	14.648	90	90	90	71.2	-136.36	7.75
f149	$Pbca$	8.940	9.316	12.297	90	90	90	73.9	-136.36	7.75
f1490	$P2_1/c$	9.283	8.961	12.263	90	90.94	90	74.2	-136.34	7.76
f131	$Pccn$	12.312	8.940	9.298	90	90	90	73.9	-136.31	7.80
f796	$Pbca$	8.952	9.301	24.722	90	90.00	90	73.5	-136.27	7.83
f116	$Pccn$	12.424	8.957	9.285	90	90	90	73.3	-136.27	7.84
f15	$P2_1$	3.916	9.143	14.837	90	90	90	71	-136.25	7.85
f3844	$C2/c$	12.748	9.307	17.538	90	104.28	90	75.1	-136.24	7.86
f873	$Pbca$	9.044	9.249	24.796	90	90	90	73	-136.23	7.87
f690	$Pccn$	24.874	8.964	9.294	90	90	90	73.1	-136.22	7.88
f100	$Pca2_1$	6.210	8.966	9.295	90	90	90	73	-136.22	7.89
f1193	$C2$	9.004	9.277	12.444	90	90.06	90	72.8	-136.20	7.90

ID[a]	Space Group	a	b	c	α	β	γ	PI[b]	E_{latt}	ΔE_{latt}
		/ Å			/ °				/ kJ mol^{-1}	
f824	$P2/c$	8.972	9.271	12.437	90	92.78	90	73.3	-136.16	7.94
f110	$Iba2$	8.967	8.967	8.967	119.72	92.21	117.75	72.9	-136.15	7.95
f1601	P-1	7.989	8.117	9.017	72.89	72.28	70.14	74	-136.10	8.00
f3074	$Pna2_1$	27.813	7.915	4.759	90	90	90	72.3	-136.09	8.01
f1728	$P2_1/c$	9.265	12.372	9.027	90	90.14	90	73.2	-136.08	8.02
f691	$P2_12_12$	12.393	9.005	9.261	90	90	90	73.3	-136.07	8.03
f284	$Pbca$	9.009	9.262	24.806	90	90	90	73.2	-136.05	8.05
f2048	$P2_12_12_1$	9.304	6.120	8.940	90	90	90	74.2	-136.05	8.06
f4235	$P2_1/c$	9.300	6.117	8.947	90	90	90	74.2	-136.02	8.08
f832	$Iba2$	24.842	8.896	9.323	90	90	90	73.4	-136.01	8.09
f799	$Pbca$	9.036	9.249	24.684	90	90	90	73.4	-136.01	8.10
f724	$Aba2$	9.323	6.271	8.843	90	90	90	72.8	-135.96	8.14
f1978	$C2/c$	6.407	9.336	17.951	90	90.05	90	70.2	-135.92	8.19
f861	$C2/c$	23.932	3.609	26.280	90	91.80	90	66.4	-135.91	8.20
f14	$P2_1/c$	12.250	3.745	22.780	90	91.91	90	72.2	-135.89	8.21
f623	$Pccn$	24.845	8.971	9.282	90	90	90	73.1	-135.89	8.22
f1069	$Pccn$	24.719	8.927	9.306	90	90	90	73.6	-135.86	8.24
f335	$P2_1/c$	3.633	18.892	8.197	90	96.29	90	67.6	-135.85	8.25
f5059	$P2_1$	7.876	4.782	14.635	90	90.03	90	68.5	-135.84	8.27
f1299	$P2_1/c$	8.310	9.230	7.128	90	108.41	90	72.8	-135.82	8.28
f399	$C2$	6.395	9.250	8.975	90	101.47	90	78.8	-135.78	8.32
f525	$Aba2$	9.289	6.317	17.760	90	90	90	72.6	-135.77	8.33
f1148	$Pca2_1$	12.436	8.874	9.327	90	90	90	73.5	-135.74	8.36
f5564	$C2$	5.624	5.624	18.030	82.71	82.71	110.42	72.6	-135.72	8.38
f5228	$P2_1/c$	5.449	35.219	5.821	90	110.58	90	72.1	-135.72	8.38
f5774	$Pbca$	9.250	7.033	31.746	90	90	90	73.3	-135.70	8.41
f1954	$P2_12_12_1$	9.184	9.234	12.383	90	90	90	72.1	-135.67	8.44
f148	$P2_12_12_1$	8.957	9.275	12.451	90	90	90	73.3	-135.65	8.46
f566	$Pbca$	9.252	7.005	15.838	90	90	90	73.7	-135.63	8.48
f2026	$P3_12_1$	9.318	9.318	22.001	90	90	120	68.3	-135.62	8.49
f5244	$Aba2$	36.451	6.343	9.340	90	90	90	69.9	-135.56	8.54
f1015	$Pbcn$	25.127	8.970	9.288	90	90	90	72.4	-135.54	8.56
f3254	$Iba2$	24.975	9.016	9.263	90	90	90	72.5	-135.54	8.57
f115	$C2/c$	12.581	8.893	9.349	90	100.23	90	73.7	-135.53	8.57
f1033	$C2/c$	25.072	9.008	9.281	90	95.98	90	72.8	-135.51	8.59
f1268	$P2_1/c$	8.858	9.317	13.156	90	109.57	90	74	-135.50	8.60
f618	$C2/c$	25.058	8.908	9.328	90	98.19	90	73.5	-135.47	8.64
f1974	$P2_1/c$	8.476	3.657	32.796	90	92.82	90	74.5	-135.46	8.64
f4828	$C2/c$	25.191	8.965	9.268	90	97.13	90	73	-135.45	8.65
f1667	$Pna2_1$	16.489	8.499	3.599	90	90	90	75	-135.44	8.67
f1029	$C2/c$	14.118	11.836	13.897	90	110.60	90	69.4	-135.43	8.67
f5000	$P2_1/c$	9.048	12.484	9.231	90	90.12	90	72.6	-135.43	8.67
f975	$P2_12_12_1$	9.019	9.260	12.341	90	90	90	73.5	-135.43	8.67
f671	$Pna2_1$	8.943	12.450	9.301	90	90	90	73.1	-135.41	8.69
f40	$P2_1/c$	6.808	8.922	8.974	90	109.31	90	73.8	-135.41	8.70
f1483	$P2_1/c$	8.485	3.668	32.894	90	94.84	90	74	-135.40	8.70
f3625	$P2_1/c$	5.140	7.222	39.673	90	42.67	90	70.9	-135.35	8.75
f1539	$C2/c$	15.723	9.214	7.002	90	90.76	90	74.5	-135.34	8.77
f596	$P2_1/c$	9.278	12.512	9.036	90	90.14	90	72.2	-135.33	8.77
f1379	$P2_1$	8.437	3.665	17.835	90	102.34	90	70.1	-135.30	8.80
f933	$Pbca$	9.040	9.245	24.870	90	90	90	73	-135.29	8.81
f763	P-1	7.148	8.552	8.812	88.51	86.91	87.85	70.1	-135.28	8.83

ID[a]	Space Group	a	b	c	α	β	γ	PI[b]	E_{latt}	ΔE_{latt}
		/ Å			/ °				/ kJ mol^{-1}	
f1350	*C*2/*c*	25.192	8.998	9.249	90.00	94.31	90.00	72.5	-135.26	8.84
f2391	*P*-1	7.169	8.666	8.904	83.97	84.35	71.09	73.1	-135.26	8.85
f1599	*Pbcn*	24.875	8.904	9.330	90	90	90	73.2	-135.22	8.88
f282	*Pbca*	9.025	9.254	24.912	90	90	90	72.9	-135.21	8.89
f835	$P2_1/c$	8.991	9.266	12.650	90	101.98	90	73.5	-135.20	8.91
f1365	$P2_1/c$	13.063	9.011	9.270	90	108.88	90	73.5	-135.17	8.93
f2740	*P*2/*c*	9.007	9.271	12.929	90	103.66	90	72	-135.16	8.95
f1394	$P2_12_12_1$	4.013	9.168	28.749	90	90.00	90	71.4	-135.15	8.96
f401	$P2_1$	4.819	9.307	6.116	90	108.77	90	79.1	-135.09	9.01
f176	$P2_1$	4.352	9.282	12.824	90	92.32	90	73.2	-135.08	9.02
f1854	$P2_1$	4.464	9.324	6.437	90	107.01	90	73.8	-135.08	9.02
f3882	*P*2/*c*	8.987	9.281	13.116	90	106.86	90	72.2	-135.08	9.03
f3390	$P2_1/c$	8.895	12.559	9.313	90	90.16	90	72.6	-135.08	9.03
f1905	*C*2/*c*	15.802	9.224	6.964	90	90.69	90	74.6	-135.07	9.04
f1345	$P2_1/c$	12.463	9.068	9.223	90	94.75	90	73	-135.07	9.04
f680	*P*2/*c*	17.427	3.582	17.943	90	97.55	90	67.9	-135.05	9.05
f1298	*Pbca*	8.973	9.290	25.259	90	90	90	72	-135.04	9.06
f556	*P*2/*c*	17.490	3.616	17.666	90	94.51	90	67.6	-135.03	9.07
f310	$P2_1/c$	3.659	16.812	8.273	90	94.81	90	74.5	-135.03	9.08
f2522	$P2_1/c$	12.810	8.998	9.266	90	104.11	90	73	-134.98	9.12
f803	*C*2/*c*	25.175	9.009	9.258	90	92.75	90	72.3	-134.98	9.12
f3496	*P*-1	7.121	9.025	9.389	74.03	79.53	69.38	69.8	-134.97	9.13
f1434	*Pbca*	9.040	9.253	24.945	90	90	90	72.5	-134.96	9.15
f2051	$P2_1/c$	12.665	8.954	9.288	90	95.36	90	72.3	-134.94	9.17
f43	*P*-1	3.626	11.452	13.200	94.78	94.04	94.72	69.4	-134.92	9.19
f439	$P2_1/c$	8.432	9.301	7.029	90	110.18	90	73.2	-134.91	9.19
f660	$P2_1/c$	5.510	18.322	5.787	90	111.86	90	69.7	-134.90	9.20
f782	$P2_1/c$	7.912	4.869	26.732	90.00	93.22	90	73.6	-134.90	9.20
f48	*R*3*c*	14.479	14.479	13.383	90	90	120	70.3	-134.90	9.20
f2819	*Pccn*	25.024	9.001	9.278	90	90	90	72.4	-134.89	9.21
f1147	Cc	7.862	12.900	10.363	90	90.71	90	72.1	-134.89	9.21
f3787	*Pccn*	25.014	9.020	9.257	90	90	90	72.5	-134.88	9.22
f1410	*Pbca*	8.952	9.315	24.941	90	90	90	73	-134.88	9.22
f673	$P2_1/c$	12.879	8.930	9.290	90	102.40	90	72.7	-134.88	9.22
f510	*C*2/*c*	9.284	8.984	25.164	90	91.51	90	72.3	-134.88	9.22
f1200	$P2_1/c$	3.644	37.298	8.151	90	96.62	90	68.6	-134.82	9.29
f1654	$P2_1/c$	8.485	3.624	33.184	90	93.66	90	74.2	-134.76	9.34
f5347	Cc	37.460	6.324	9.365	90	90	90	67.9	-134.74	9.37
f2671	*C*2/*c*	9.331	8.897	12.409	90	91.52	90	73.5	-134.73	9.38
f4544	*C*2/*c*	25.254	8.998	9.275	90	96.23	90	72.3	-134.70	9.40
f336	*Fdd*2	33.379	15.672	3.731	90	90	90	77.9	-134.70	9.41
f2495	$P4_12_12$	9.160	9.160	12.551	90	90	90	71.9	-134.69	9.42
f137	*C*2/*c*	7.797	8.732	15.104	90	102.41	90	75.4	-134.66	9.44
f1170	*C*2/*c*	25.080	8.923	9.318	90	93.30	90	72.8	-134.65	9.45
f3643	$Pna2_1$	9.286	8.950	12.464	90	90	90	73.1	-134.63	9.47
f1328	*P*-1	7.603	7.838	9.277	84.88	85.71	71.59	72.7	-134.62	9.48
f544	*P*-1	8.391	8.424	9.022	68.81	69.14	66.82	71.4	-134.60	9.50
f1522	$P2_1/c$	9.290	8.976	12.689	90	91.32	90	71.6	-134.60	9.50
f440	*Pc*	3.663	8.980	8.068	90	97.65	90	72	-134.60	9.51
f447	$P2_1/c$	8.999	9.272	13.190	90	107.86	90	72.3	-134.58	9.52
f5301	$P2_1/c$	7.032	18.549	8.922	90	109.40	90	68.8	-134.58	9.52
f427	$P2_1/c$	12.544	8.919	9.289	90	92.17	90	72.9	-134.58	9.53

ID[a]	Space Group	a	b	c	α	β	γ	PI[b]	E_{latt}	ΔE_{latt}
		/ Å			/ °				/ kJ mol^{-1}	
f468	$P2_1/c$	9.848	9.348	11.156	90	93.67	90	74	-134.55	9.55
f4419	*P*1	3.657	8.086	9.034	88.30	92.05	82.84	71.6	-134.52	9.58
f3245	*Pbca*	8.905	9.333	24.860	90	90.00	90	73.3	-134.52	9.59
f1196	$P2_1/c$	15.385	4.262	17.284	90	113.63	90	72.8	-134.51	9.59
f3785	*P*2/*c*	9.328	8.906	12.401	90	91.33	90	73.6	-134.51	9.59
f4217	*Fdd*2	73.267	6.301	9.283	90	90.00	90	70.4	-134.50	9.60
f1931	$P2_1/c$	12.422	9.074	9.217	90	92.27	90	72.9	-134.49	9.62
f2143	$P2_1/c$	7.612	15.938	9.278	90	111.03	90	72.1	-134.47	9.63
f1119	*P*-1	7.408	8.274	9.320	79.07	78.59	69.23	73	-134.45	9.65
f1445	$P4_3$	9.215	9.215	12.568	90	90	90	71	-134.44	9.66
f822	*Cc*	5.477	18.860	5.790	90	111.93	90	67.8	-134.42	9.68
f754	$P2_1/c$	8.623	9.306	6.884	90	111.28	90	73.4	-134.42	9.69
f3269	*Pbcn*	8.950	9.284	24.935	90	90	90	73	-134.42	9.69
f5109	*P*-1	7.699	8.455	9.267	79.91	68.95	73.46	70.3	-134.37	9.73
f2786	$P2_1/c$	4.657	31.814	7.227	90	99.98	90	71.7	-134.36	9.74
f3136	*Pbca*	9.311	6.978	32.749	90	90	90	71.2	-134.34	9.77
f2556	*Pbca*	9.306	12.430	17.741	90	90	90	73.8	-134.33	9.77
f2071	*Fdd*2	36.888	12.189	9.455	90	90	90	71.1	-134.23	9.87
f2538	*P*-1	7.166	8.914	9.536	72.30	80.03	69.67	69.6	-134.20	9.91
f3505	$P2_1/c$	8.946	9.302	12.544	90	90.22	90	72.5	-134.14	9.96
f2198	$Pna2_1$	9.324	8.909	12.676	90	90	90	72	-134.13	9.97
f893	*P*-1	5.362	10.192	11.279	116.14	90.51	94.41	68.2	-134.13	9.98
f2266 (**fC-II**)	*Pccn*	15.963	16.183	9.376	90	90	90	62.7	-127.03	17.07

[a]Structure ID: f – 5-flucytosine and rank CrystalPredictor. The CASTEP minimised structures were checked for higher symmetry using PLATON.[16] **fC-I** and **fC-II** – isostructural with **C-I** and **C-II**. [b]Packing Index (%) calculated using PLATON.

1.2.3. Cytosine/5-Flucytosine Low-Energy Structures

Table S4. Hypothetical Low-Energy Crystal Structures of Cytosine/5-Flucytosine Anhydrate 1:1 "Cocrystals" (PBE-TS and PBE-D2 energies). Experimental structures are highlighted in green.

ID[a]	Space	a	b	c	α	β	γ	PI[b]	E_{latt}	ΔE_{latt}
	Group	/ Å			/ °				/ kJ mol^{-1}	
cf3755 (**CF-I**)	$P2_12_12_1$	5.587	7.602	23.549	90	90	90	73.20	-311.22	0.00
cf21	$P2_1/c$	9.499	3.928	25.759	90	90.17	90	76.30	-311.04	0.18
cf207 (**CF-II**)	Pn	3.911	9.391	13.123	90	90.31	90	76.50	-307.43	3.78
cf980	$P2_1/c$	8.170	15.934	8.417	90	112.53	90	72.50	-307.06	4.16
cf40 (**CF-II**)	$P2_1$	3.979	9.391	13.023	90	91.70	90	75.60	-306.97	4.25
cf5 (**CF-II**)	$P2_1/c$	7.743	9.420	13.553	90	105.00	90	77.00	-306.83	4.39
cf6028 (**CF-II**)	$P2_1/c$	7.801	9.365	13.664	90	105.84	90	76.80	-306.29	4.93
cf42 (**CF-II**)	P-1	3.821	9.401	13.362	89.10	87.81	87.18	76.80	-306.01	5.21
cf4 (**CF-II**)	$P2_1/n$	7.778	9.361	13.743	90	106.08	90	76.80	-306.01	5.21
cf16 (**CF-II**)	$P2_1/c$	7.799	9.370	13.668	90	105.58	90	76.60	-305.85	5.37
cf1809 (**CF-II**)	P-1	3.806	9.400	13.386	89.32	87.50	87.02	77.00	-305.68	5.54
cf50 (**CF-II**)	$P2_1$	3.944	9.384	13.058	90	90.35	90	76.20	-305.65	5.56
cf3 (**CF-II**)	$P2_1/c$	7.760	9.372	13.658	90	105.15	90	76.80	-305.57	5.65
cf11 (**CF-II**)	$P2_1/n$	7.686	9.404	13.645	90	104.37	90	77.00	-305.55	5.66
cf87 (**CF-II**)	P-1	3.797	9.404	13.328	88.94	86.78	87.79	77.60	-305.46	5.76
cf1957 (**CF-II**)	$P2_1/n$	7.737	9.394	13.603	90	104.70	90	76.90	-305.16	6.06
cf33	$P2_1/c$	9.374	4.079	26.020	90	96.61	90	74.20	-303.18	8.04
cf6888	$P2_1/n$	9.287	9.518	11.063	90	97.17	90	76.10	-302.27	8.95
cf3232	$C2/c$	17.520	6.894	16.950	90	102.16	90	73.20	-302.15	9.06
cf1590	$P2_1/c$	13.573	3.684	21.121	90	91.21	90	69.40	-301.23	9.99
cf101	$P2_1/n$	3.883	26.552	9.419	90	92.54	90	75.90	-301.02	10.19
cf1808	$P2_1/n$	3.903	26.510	9.405	90	93.36	90	75.70	-301.01	10.21
cf1339	$P2_1/n$	3.882	26.550	9.429	90	92.18	90	75.80	-300.62	10.60
cf2046	$P2_1/c$	7.714	9.456	13.601	90	99.80	90	75.50	-300.60	10.62
cf485	$P2_1/n$	12.056	5.022	16.960	90	106.43	90	74.60	-300.32	10.90
cf5379	$P2_1/c$	7.693	9.446	13.658	90	99.56	90	75.40	-300.24	10.97
cf369	$P2_1/n$	9.162	9.321	11.652	90	100.27	90	75.50	-299.80	11.42
cf1995	$P2_1/n$	3.901	26.579	9.388	90	90.34	90	75.80	-299.69	11.52
cf2485	$P2_1/n$	9.167	9.324	11.644	90	100.39	90	75.60	-299.57	11.64
cf5663	P-1	3.660	11.322	12.540	89.19	87.62	82.08	71.60	-299.31	11.91
cf5180	P-1	3.658	11.338	12.561	88.99	87.44	82.14	71.30	-299.21	12.01
cf114	$P2_1/c$	15.999	3.852	17.302	90	116.35	90	77.00	-299.20	12.02
cf6381	P-1	3.657	11.344	12.580	88.42	87.47	81.90	71.20	-299.18	12.03
cf4622	$C2/c$	18.451	3.958	27.672	90	100.96	90	74.00	-298.76	12.46
cf32	$P2_1$	9.215	4.043	13.686	90	90.41	90	71.90	-298.72	12.49
cf27	$P2_1$	9.244	3.994	13.782	90	91.88	90	72.00	-298.15	13.07
cf4039	$P2_12_12_1$	4.297	9.388	24.772	90	90	90	73.60	-297.98	13.23
cf29	$P2_1/c$	9.226	3.731	28.598	90	95.09	90	75.20	-297.77	13.45
cf19	$P2_1/c$	9.217	3.750	28.655	90	96.05	90	74.90	-297.76	13.46
cf172	C2/c	18.470	3.901	28.047	90	100.69	90	74.00	-297.66	13.55
cf1092	$P2_1/c$	9.223	3.731	28.601	90	95.04	90	75.30	-297.59	13.63
cf1037	C2/c	12.874	9.374	17.154	90	106.45	90	74.30	-297.49	13.73
cf3236	$P2_12_12_1$	4.210	9.299	25.996	90	90	90	72.20	-297.37	13.85
cf948	$P2_1/c$	3.727	9.237	28.507	90	93.67	90	75.40	-297.28	13.94
cf3609	$P2_1$	4.049	13.845	9.221	90	91.28	90	71.00	-296.97	14.25
cf54	$P2_1$	4.065	9.224	13.716	90	90.63	90	71.20	-296.74	14.48
cf811	$P2_1$	4.068	13.761	9.261	90	91.26	90	70.50	-296.40	14.81

ID[a]	Space Group	a	b	c	α	β	γ	PI[b]	E_{latt}	ΔE_{latt}
		/ Å			/ °				/ kJ mol^{-1}	
cf6652	*P*-1	7.104	8.517	8.584	90.92	91.49	96.54	71.30	-296.30	14.92
cf2717	*P*-1	7.085	8.468	8.618	90.88	91.30	96.24	71.50	-296.25	14.97
cf5714	$P2_1/c$	3.802	9.272	34.892	90	127.22	90	75.20	-296.16	15.05
cf1774	*P*-1	7.477	8.109	8.554	85.04	71.81	84.25	75.20	-296.02	15.20
cf6907	*P*-1	7.460	8.122	8.579	85.31	71.64	84.49	75.10	-295.89	15.33
cf2469	*P*-1	7.463	8.121	8.624	85.14	71.42	84.56	74.90	-295.85	15.37
cf2950	*P*-1	3.702	9.278	15.877	105.56	91.97	91.65	70.30	-295.78	15.44
cf222	*C*2/*c*	18.347	3.728	29.287	90	99.46	90	74.50	-295.77	15.45
cf4465	*P*-1	7.124	8.533	8.557	90.91	91.98	96.45	71.20	-295.68	15.54
cf1911	*Pbca*	14.015	9.744	14.203	90	90	90	75.80	-295.58	15.64
cf2688	$P2_1/c$	3.735	28.912	9.260	90	90.88	90	73.90	-295.45	15.77
cf1952	$Pna2_1$	9.290	27.029	4.119	90	90	90	71.00	-294.92	16.30
cf93	$Pna2_1$	28.878	9.240	3.759	90	90	90	73.40	-294.75	16.46
cf3263	$Pna2_1$	9.326	26.820	4.116	90	90	90	71.30	-294.63	16.59
cf445	*P*-1	6.694	7.919	9.508	88.04	84.31	78.90	75.00	-294.03	17.19
cf1528	$P2_1$	8.474	3.721	15.767	90	95.47	90	74.10	-294.00	17.22
cf298	*I*2	15.933	4.064	17.653	90	115.34	90	70.90	-293.99	17.22
cf76	$P2_1/c$	16.206	3.749	17.776	90	115.70	90	75.70	-293.93	17.29
cf1495	*P*-1	6.700	7.945	9.514	88.02	84.05	78.23	74.90	-293.89	17.33
cf271	*P*-1	6.670	7.954	9.516	88.71	85.94	78.53	74.70	-293.62	17.60
cf4233	*P*-1	7.116	8.139	9.449	82.12	80.65	66.07	75.00	-293.07	18.15
cf1060	*C*2/*c*	18.387	3.654	32.326	90	103.12	90	69.70	-293.07	18.15
cf1974	$P2_1/c$	8.078	6.627	18.690	90	90.88	90	73.60	-292.87	18.35
cf1044	$P2_1/c$	8.632	3.692	31.224	90	96.89	90	74.50	-292.85	18.37
cf4705	*I*2/*c*	12.610	9.307	17.154	90	100.49	90	74.70	-292.84	18.38
cf3862	$P2_1/c$	8.082	6.631	18.694	90	90.82	90	73.50	-292.75	18.47
cf127	*P*-1	7.075	8.174	9.469	82.42	81.03	65.49	75.20	-292.70	18.52
cf3074	$P2_1/c$	9.492	6.670	15.499	90	90.95	90	75.00	-292.66	18.55
cf1792	$P2_1/c$	7.261	15.812	9.249	90	111.12	90	74.50	-292.33	18.88
cf1433	*P*-1	7.128	8.102	9.431	82.54	81.35	67.44	74.50	-291.88	19.34
cf1255	*Pbca*	9.573	13.950	14.712	90	90	90	75.10	-291.57	19.65
cf876	$Pna2_1$	28.393	9.262	3.950	90	90	90	70.70	-291.53	19.69
ct443	*P*-1	7.141	8.141	9.432	81.88	80.19	67.01	74.50	-291.45	19.77

[a]Structure ID: cf – cytosine/5-flucytosine and rank CrystalPredictor. The CASTEP minimised structures were checked for higher symmetry using PLATON.[16] [b]Packing Index (%) calculated using PLATON.

1.3. Representation of the Experimental Structures

The computational models were successful in reproducing the experimental anhydrate and hydrate structures of cytosine (Table S5 taken from ref. 17) and 5-flucytosine (Table S6 taken from ref. 17).

The computationally generated low energy structures were compared using the Solid Form module of Mercury to determine the root mean square deviation of the non-hydrogen atoms in a cluster of 15 molecules (rmsd15).[18]

1.3.1. Cytosine

Table S5. Quality of Representation of the Experimental Cytosine Structures.

Method	Lattice parameters (cell vectors/Å, angles/°)						density	rmsd$_{15}$
	a	*b*	*c*	α	β	γ	(g cm^{-3})	(Å)[18]
Calc., PBE-TS, 0 K	12.846	9.486	3.783	90	90	90	1.601	–
Exptl., C-I, CYTSIN01, RT	13.044	9.496	3.814	90	90	90	1.562	0.098
Calc., PBE-TS, 0 K	14.999	14.949	9.292	90	90	90	1.418	–
Exptl., C-II, CYTSIN02, RT	15.104	15.121	9.295	90	90	90	1.391	0.068
Calc., PBE-TS, 0 K	7.780	9.758	7.388	90	99.27	90	1.549	–
Exptl., cH1, CYTOSM, RT	7.801	9.844	7.683	90	99.70	90	1.475	0.112
Exptl., cH1, CYTOSM02, RT	7.783	9.825	7.668	90	99.57	90	1.483	0.104
Exptl., cH1, CYTOSM11, RT	7.783	9.825	7.668	90	99.57	90	1.483	0.104
Exptl., cH1, CYTOSM13, 100 K	7.718	9.814	7.522	90	100.48	90	1.531	0.080
Exptl., cH1, CYTOSM03, 97 K	7.728	9.817	7.520	90	100.50	90	1.529	0.079
Exptl., cH1, CYTOSM12, 90 K	7.716	9.834	7.513	90	100.52	90	1.530	0.081
Exptl., cH1, CYTOSM04, 82 K	7.713	9.830	7.505	90	100.52	90	4.592	0.080

1.3.2. 5-Flucytosine

Table S6. Quality of Representation of the Experimental 5-Flucytosine Anhydrate and Monohydrate Structures.

Method	Lattice parameters (cell vectors/Å, angles/°)						density	rmsd$_{15}$
	a	*b*	*c*	α	β	γ	(g cm^{-3})	(Å)[18]
Calc., PBE-TS, 0 K	6.688	6.688	23.354	90	90	90	1.642	–
Exptl., F-I, MEBQEQ01, 150 K	6.639	6.639	23.471	90	90	90	1.658	0.05
Calc., PBE-TS, 0 K	4.080	9.522	12.896	90	91.78	90	1.712	–
Exptl., F-II, MEBQEQ, 150 K	4.063	9.521	12.739	90	92.99	90	1.743	0.08
Calc., PBE-TS, 0 K	7.406	9.427	17.570	90	99.06	90	1.613	–
Exptl., fH1-I, BIRMEU, RT	7.562	9.390	21.361	90	125.13	90	1.575	0.08
Exptl., fH1-I, BIRMEU01, RT	7.514	9.424	17.692	90	99.16	90	1.580	0.07
Exptl., fH1-I, BIRMEU02, 150 K	7.387	9.394	17.579	90	98.61	90	1.620	0.06
Calc., PBE-TS, 0 K	4.133	8.213	9.917	109.24	100.52	97.31	1.596	–
Exptl., fH1-II, BIRMEU03, 150 K	4.103	8.273	9.919	110.04	100.46	96.71	1.601	0.06
Calc., PBE-TS, 0 K	14.621	12.432	13.739	90	115.15	90	1.623	–
Exptl., Hemihydrate, DUKWIQ, 173 K	14.704	12.455	13.792	90	115.47	90	1.609	0.04
Calc., PBE-TS, 0 K	12.185	9.445	13.898	90	111.88	90	1.558	–
Exptl., Hemipentahydrate, MEBQUG, 150 K	12.238	9.425	13.873	90	111.39	90	1.553	0.09

1.4. Selected Computed Low-Energy Structures (Possible Polymorphs)

1.4.1. Cytosine: c123

```
TITL c123
CELL  1.54180    9.4182    3.7288   26.2602    90.000    90.287    90.000
ZERR       8    0.0000    0.0000    0.0000     0.000     0.000     0.000
LATT   1
SYMM           - X ,    0.50000 + Y ,    0.50000 - Z
SFAC  C  H  N  O
C    1  0.76433  0.96496  0.19062  11.00000   0.0500
C    1  0.51359  0.92483  0.18299  11.00000   0.0500
C    1  0.65295  1.19121  0.11688  11.00000   0.0500
C    1  0.26262  0.59065  0.06231  11.00000   0.0500
C    1  0.12925  0.69788  0.04168  11.00000   0.0500
C    1  0.01164  0.61044  0.06882  11.00000   0.0500
C    1  0.15316  0.33793  0.13432  11.00000   0.0500
C    1  0.63168  0.85621  0.21125  11.00000   0.0500
H    2  0.42774  1.19543  0.12117 11.00000 -1.20000
H    2  0.87780  0.82376  0.25414 11.00000 -1.20000
H    2  0.40600  0.85437  0.19429 11.00000 -1.20000
H    2  0.98153  0.98423  0.20181 11.00000 -1.20000
H    2  0.62716  0.72376  0.24787 11.00000 -1.20000
H    2  0.47920  0.57042  0.05125 11.00000 -1.20000
H    2  0.37641  0.74198 -0.00032 11.00000 -1.20000
H    2  0.12300  0.84031  0.00573 11.00000 -1.20000
H    2 -0.09675  0.66868  0.05671 11.00000 -1.20000
H    2 -0.07290  0.32749  0.12951 11.00000 -1.20000
N    3  0.52321  1.09116  0.13726  11.00000   0.0500
N    3  0.88474  0.90915  0.21695  11.00000   0.0500
N    3  0.77279  1.12333  0.14445  11.00000   0.0500
N    3  0.02266  0.43588  0.11391  11.00000   0.0500
N    3  0.27225  0.41765  0.10745  11.00000   0.0500
N    3  0.38316  0.66178  0.03708  11.00000   0.0500
O    4  0.65596  1.34640  0.07410  11.00000   0.0500
O    4  0.15756  0.17188  0.17637  11.00000   0.0500
END
```

1.4.2. 5-Flucytosine: f3194

```
TITL f3194
CELL  1.54180   17.4613   6.9328   17.0909   90.000   101.689   90.000
ZERR      16   0.0000   0.0000   0.0000   0.000   0.000   0.000
LATT   7
SYMM          - X ,           Y ,   0.50000 - Z
SFAC  C  H  F  N  O
C    1  0.16228  0.39782  0.32903  11.00000   0.0500
C    1  0.28985  0.52959  0.33226  11.00000   0.0500
C    1  0.22191  0.45955  0.28904  11.00000   0.0500
C    1  0.24131  0.49106  0.45311  11.00000   0.0500
C    1  0.45457  0.30491  0.07635  11.00000   0.0500
C    1  0.53031  0.24192  0.11519  11.00000   0.0500
C    1  0.58248  0.18258  0.07136  11.00000   0.0500
C    1  0.49004  0.25358 -0.04866  11.00000   0.0500
H    2  0.35225  0.60598  0.44580 11.00000 -1.20000
H    2  0.05279  0.28285  0.32121 11.00000 -1.20000
H    2  0.08245  0.31308  0.22795 11.00000 -1.20000
H    2  0.33874  0.57447  0.30606 11.00000 -1.20000
H    2  0.60575  0.14535 -0.04336 11.00000 -1.20000
H    2  0.34547  0.40917  0.08568 11.00000 -1.20000
H    2  0.41296  0.36280  0.17739 11.00000 -1.20000
H    2  0.64064  0.12777  0.09687 11.00000 -1.20000
F    3  0.21037  0.44589  0.20753  11.00000   0.0500
F    3  0.55014  0.24192  0.19697  11.00000   0.0500
N    4  0.29918  0.54529  0.41256  11.00000   0.0500
N    4  0.09565  0.31821  0.28937  11.00000   0.0500
N    4  0.17358  0.41742  0.40893  11.00000   0.0500
N    4  0.56241  0.18819 -0.00947  11.00000   0.0500
N    4  0.43731  0.30791 -0.00431  11.00000   0.0500
N    4  0.40030  0.36252  0.11651  11.00000   0.0500
O    5  0.25236  0.50958  0.52772  11.00000   0.0500
O    5  0.47472  0.26205 -0.12428  11.00000   0.0500
END
```

1.4.3. Cytosine/5-Flucytosine: ss21

```
TITL ss21
CELL  1.54180   9.4985   3.9283   25.7589   90.000   90.169   90.000
ZERR      4   0.0000   0.0000   0.0000    0.000    0.000    0.000
LATT   1
SYMM          - X ,    0.50000 + Y ,    0.50000 - Z
SFAC  C  H  F  N  O
C   1  0.26471  0.56161  0.06618  11.00000   0.0500
C   1  0.13393  0.68585  0.04729  11.00000   0.0500
C   1  0.01279  0.59921  0.07155  11.00000   0.0500
C   1  0.14689  0.29487  0.13559  11.00000   0.0500
C   1  0.76338  0.93069  0.19064  11.00000   0.0500
C   1  0.51479  0.87993  0.18420  11.00000   0.0500
C   1  0.63395  0.80778  0.21123  11.00000   0.0500
C   1  0.64694  1.18067  0.11940  11.00000   0.0500
H   2 -0.07716  0.30048  0.12950 11.00000 -1.20000
H   2  0.47977  0.53994  0.05471 11.00000 -1.20000
H   2  0.37698  0.73514  0.00430 11.00000 -1.20000
H   2 -0.09151  0.67676  0.05878 11.00000 -1.20000
H   2  0.42483  1.16232  0.12455 11.00000 -1.20000
H   2  0.97930  0.95046  0.20105 11.00000 -1.20000
H   2  0.87918  0.76998  0.25310 11.00000 -1.20000
H   2  0.41005  0.79561  0.19554 11.00000 -1.20000
H   2  0.63254  0.65986  0.24668 11.00000 -1.20000
F   3  0.13614  0.88858  0.00430  11.00000   0.0500
N   4  0.01967  0.40307  0.11493  11.00000   0.0500
N   4  0.26785  0.37348  0.10992  11.00000   0.0500
N   4  0.38412  0.63295  0.04088  11.00000   0.0500
N   4  0.52038  1.06513  0.13986  11.00000   0.0500
N   4  0.88399  0.86902  0.21611  11.00000   0.0500
N   4  0.76818  1.10693  0.14554  11.00000   0.0500
O   5  0.14802  0.11926  0.17686  11.00000   0.0500
O   5  0.64527  1.35355  0.07809  11.00000   0.0500
END
```

2. EXPERIMENTAL

2.1. Preparation of Anhydrate Solid Solutions

Anhydrate **CF-I** was obtained by slurring the two compounds in 1-butanol in between 10 and 40 °C for two weeks.

Anhydrate **CF-II** was prepared starting from the monohydrate I solid solution. **C-I** and **F-II** were stirred in water in the temperature range from 10 to 20 °C for 72 hours. The resulting monohydrate was filtered and dried at 43 % RH (over saturated a saturated K_2CO_3 solution). Dehydration of the monohydrate over P_2O_5 (0% RH) at 25 °C resulted in **CF-II**. (Heating **CF-II** to 230 °C for 30 minutes or dehydrating the monohydrate in a sealed DSC pan leads to **CF-I**.)

2.2. Methodology

2.2.1. Thermal Analysis

A Reichert Thermovar polarisation microscope, equipped with a Kofler hot-stage (Reichert, A), was used for *hot-stage thermal microscopy (HSM)* investigations. Photographs were taken with an Olympus DP71 digital camera (Olympus, D).

Differential Scanning Calorimetry (DSC) thermograms were recorded with a DSC 7 (Perkin-Elmer Norwalk, Ct., USA) and controlled by the Pyris 2.0 software. Using a UM3 ultramicrobalance (Mettler, Greifensee, CH), samples of approximately 2 – 3 mg were weighed into perforated aluminium pans. The samples were heated using rates in between 5 and 20 °C min^{-1} with dry nitrogen as the purge gas (purge: 20 mL min^{-1}). The instrument was calibrated for temperature with pure benzophenone (mp 48.0 °C) and caffeine (236.2 °C), and the energy calibration was performed with indium (mp 156.6 °C, heat of fusion 28.45 J g^{-1}). The errors on the stated temperatures (extrapolated onset temperatures) and enthalpy values were calculated at the 95% confidence intervals (CI) and are based on three measurements.

Thermogravimetric Analysis (TGA) was carried out with a TGA7 system (Perkin-Elmer, Norwalk, CT, USA) using the Pyris 2.0 Software. Approximately 4 – 6 mg of sample was weighed into a platinum pan. Two-point calibration of the temperature was performed with ferromagnetic materials (Alumel and Ni, Curie-point standards, Perkin-Elmer). A heating rate of 5 °C min^{-1} was applied and dry nitrogen was used as a purge gas (sample purge: 20 mL min^{-1}, balance purge: 40 mL min^{-1}).

2.2.2. Infrared Spectroscopy

Infrared spectra were recorded with a diamond ATR (PIKE GaldiATR) crystal on a Bruker Vertex 70 spectrometer (Bruker Analytische Messtechnik GmbH, D). The spectra were recorded in the range of 4000 to 30 cm^{-1} with an instrument resolution of 2 cm^{-1} (256 scans per spectrum).

2.2.3. Powder X-ray Diffraction

Powder X-ray diffraction (PXRD) patterns were obtained using an X'Pert PRO diffractometer (PANalytical, Almelo, NL) equipped with a θ/θ coupled goniometer in transmission geometry, programmable XYZ stage with well plate holder, Cu-$K_{\alpha1,2}$ radiation source with a focussing, a 0.5° divergence slit and a 0.02° Soller slit collimator on the incident beam side, a 2 mm antiscattering slit and a 0.02° Soller slit collimator on the diffracted beam side mirror and a solid state PIXcel detector. The patterns were recorded at a tube voltage of 40 kV and tube current of 40 mA, applying a step size of 2θ = 0.013° with 400 s per step in the 2θ range between 2° and 70°.

2.3. Solvent Screen

The experimental screen for mixed cytosine/5-flucytosine solid forms encompassed Crystal 16® cycling experiments, slurry experiments in selected organic solvents, dehydration and sublimation experiments.

2.3.1. Crystal16® Cycling Experiments

CF-II (10 – 15 mg) and solvents (1.0 mL) were dispensed into 1.8 mL vials. The vials were transferred to a Crystal16™ parallel crystalliser, equipped with programmable heating/cooling, magnetic stirring and turbidity sensors. The suspensions were stirred at 900 rpm at 5 °C for 30 minutes, heated to X °C at 0.1 °C min^{-1}, equilibrated for another 10 minutes, then cooled to 5 °C at 0.1 °C min^{-1} under stirring. The cycle was repeated a second time with stirring only upon heating. The solid products were isolated by filtration and analysed with PXRD (Table S7).

Table S7. Summary of Cytosine/5-Flucytosine Cycling Experiments.

Solvent	Temperature Range / °C	Solid Form[a]
methanol	5 – 50	**S-MeOH** > **H1**
ethanol	5 – 30	**S-EtOH**
1-propanol	5 – 65	**CF-II**
2-propanol	5 – 65	**CF-I** and **CF-II**
1-butanol	5 – 65	**CF-I** and **CF-II**
iso-butanol	5 – 65	**CF-II**
1-pentanol	5 – 90	**CF-I** and **CF-II**
iso-pentanol	5 – 90	**CF-I** and **CF-II**
acetone	5 – 50	**CF-II**
2-butanone	5 – 65	**CF-II**
cyclohexanone	5 – 110	**CF-I** and **CF-II**
ethyl acetate	5 – 65	**CF-II**
butyl acetate	5 – 110	**CF-II**
methyl isobutyl ketone	5 – 90	**CF-II**
dimethyl sulfoxide	5 – 90	**S-DMSO**
dimethyl formamide	5 – 110	**S-DMF**
diethyl ether	5 – 30	**CF-I** and **CF-II**
diisopropyl ether	5 – 50	**CF-I** and **CF-II**
methyl t-butyl ether	5 – 30	**H1** and **CF-II**
1,4-dioxane	5 – 65	**CF-II**
tetrahydrofuran	5 – 50	**CF-II**
acetonitrile	5 – 65	**CF-II**
nitromethane	5 – 90	**CF-II**
dichloromethane	5 – 30	**CF-II**
chloroform	5 – 50	**CF-I** and **CF-II**
carbon tetrachloride	5 – 50	**CF-II**
toluene	5 – 110	**CF-II** > **CF-I**
xylene	5 – 90	**CF-II**
hexane	5 – 50	**CF-II**
cyclohexane	5 – 50	**CF-II**

[a]H1 – monohydrate, S-MeOH – methanol solvate, S-EtOH – ethanol solvate, S-DMF – dimethyl formamide solvate, S-DMSO – dimethyl sulfoxide solvate.

2.3.2. Slurry Experiments

Suspensions of cytosine (**C-I**) and 5-flucytosine (**F-I** and **F-II** mixture) were prepared in methanol, ethanol, dimethyl formamide, dimethyl sulfoxide and 1-butanol and then stirred in the temperature range from 10 to 30 °C (40 °C – 1BuOH) for at least 96 hours. The wet-cakes were analysed by PXRD (measured between two mylar foils to prevent solvent loss). The solvate stoichiometry was determined with TGA.

Table S8. Summary of Cytosine/5-Flucytosine Slurry Experiments.

Solvent	Starting Forms	Temperature Range / °C	Solid Form
methanol	**C-I** + **F-I/F-II**	10 – 30	**S-MeOH** > **H1**
ethanol	**C-I** + **F-I/F-II**	10 – 30	**S-EtOH** > **CF-II**
DMF	**C-I** + **F-I/F-II**	10 – 30	**S-DMF**
DMSO	**C-I** + **F-I/F-II**	10 – 30	**S-DMSO** > **CF-II**
1-butanol	**C-I** + **F-I**	10 – 40	**CF-I**
1-butanol	**C-I** + **F-II**	10 – 40	**CF-I** > **CF-II**
1-butanol	**C-I** + **F-I/F-II**	10 – 40	**CF-I**

2.4. Dehydration Experiments

Dehydration studies of the monohydrate solid solution were performed and the resulting product was analysed with PXRD and TGA (Table S13).

Table S9. Dehydration Studies of Cytosine/F-Flucytosine Monohydrate I. Given are the Drying Conditions and Products.

Temperature	Solid form
Temperature, 0% RH	
25 °C	**CF-II**
40 °C	**CF-II**
60 °C	**CF-II >> CF-I**
Temperature, ambient RH	
40 °C	**CF-II**
60 °C	**CF-II >> CF-I**
Vacuum	
20 °C	**CF-II**
0 °C	**CF-II**
–20 °C	**CF-II**
DSC, closed	
170 °C	**CF-I**

2.5. Sublimation Experiments

Sublimation experiments of the solid solutions lead to phase separation ("purification") and 5-flucytosine single crystals were obtained (Figure S1).

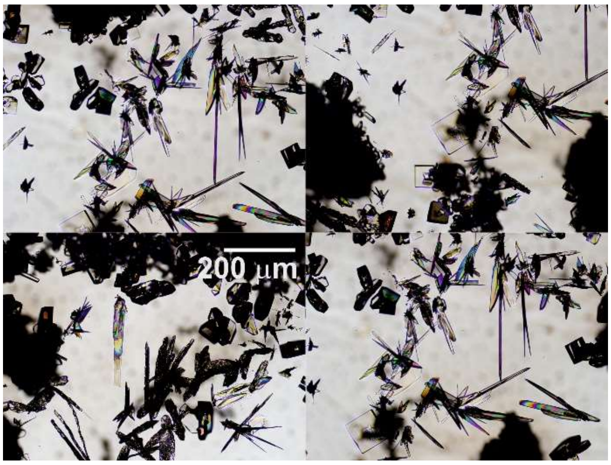

Figure S1. Cytosine/5-Flucytosine sublimation experiments at 245 °C resulting in 5-flucytosine single crystals.

2.6. Structure Determination: Simulated Annealing and Rietveld Refinement

The PXRD patterns, recorded at 25 °C, were indexed using the first twenty peaks with DICVOL04 and the space group was determined based on a statistical assessment of systematic absences,[19] as implemented in the DASH structure solution package.[20]

2.6.1. Solid Solution Anhydrate I (CF-I)

Anhydrate **CF-I** indexed to a tetragonal unit cell, $P4_12_12$, with Z′=1. The data were background subtracted and truncated to 50° 2θ for Pawley fitting.[52] Simulated annealing was used to optimise the cytosine/5-flucytosine model against the diffraction data set (75 reflections) in direct space. The internal coordinate (Z-matrix) description was derived from the PBE-TS optimised structure, with N–H distances normalized to 0.9 Å and C–H distances to 0.95 Å. The structure was solved using 200 simulated annealing runs of 2.5×10^7 moves per run as implemented in DASH, allowing 6 external degrees of freedom. The best solutions returned a χ^2 ratio of ca. 2.43 (profile χ^2/ Pawley χ^2). A restrained Rietveld refinement was carried out using the best solution returned from the simulated annealing in TOPAS Academic V5.[21] The background was modeled by a set of consecutive points with refineable intensities. The isotropic temperature factor (B_{iso}) for non-hydrogen atoms was set to 3.25 and for hydrogen atoms to 4.0. The final refinement included a total of 74 parameters (20 profile, 2 cell, 1 scale, 8 preferred orientation, 1 occupancy factor, 42 positon). The site oppupancies for H13 and F14 were refined as occ(H13) + occ(F14) = 1. The converged occ values were not significantly

different from the used starting ratio. One flatten restraint and 39 distance and angle restraints were applied. The refinement converged at R_{wp} = 6.29%, R_{exp} = 3.17%, R_p = 4.45%.

2.6.2. Solid Solution Anhydrate II (CF-II)

Anhydrate **CF-II** indexed to a monoclinic unit cell, $P2_1/n$, with Z'=1. The data were background subtracted and truncated to 52.2° 2θ for Pawley fitting.[52] Simulated annealing was used to optimise the cytosine/5-flucytosine model against the diffraction data set (95 reflections) in direct space. The internal coordinate (Z-matrix) description was derived from the PBE-TS optimised structure, with N–H distances normalized to 0.9 Å and C–H distances to 0.95 Å. The structure was solved using 200 simulated annealing runs of 2.5 $\times$ 10^7 moves per run as implemented in DASH, allowing 6 external degrees of freedom. The best solutions returned a χ^2 ratio of ca. 2.41 (profile χ^2/ Pawley χ^2). A restrained Rietveld refinement was carried out using the best solution returned from the simulated annealing in TOPAS Academic V5.[21] The background was modeled by a set of consecutive points with refineable intensities. The isotropic temperature factor (B_{iso}) for non-hydrogen atoms was set to 3.25 and for hydrogen atoms to 4.0. The final refinement included a total of 69 parameters (20 profile, 4 cell, 1 scale, 1 preferred orientation, 1 occupancy factor, 42 positon). The site oppupancies for H13 and F14 were refined as occ(H13) + occ(F14) = 1. The converged occ values were not significantly different from the used starting ratio. One flatten restraint and 39 distance and angle restraints were applied. The refinement converged at R_{wp} = 6.05%, R_{exp} = 3.34%, R_p = 4.41%.

Cell parameters for **CF-I** and **CF-II**, details of the data collection and a list of atomic parameters can be found in Tables S10-S12. Observed and calculated PXRD patterns are shown in Figure S2.

Table S10. Crystallographic Data for Anhydrates **CF-I** and **CF-II**.

	**CF-I**	**CF-II**
Crystal system, space group	*Tetragonal,* $P4_12_12$	*Monoclinic,* $P\,2_1/n$
Formula	$C_4H_{4.393}N_3OF_{0.607}$	$C_4H_{4.359}N_3OF_{0.641}$
a, Å	6.67372(5)	4.00808(9)
b, Å	6.67372(5)	9.43931(11)
c, Å	23.6290(3)	13.0306(2)
β, °	90	91.179(2)
Z	8	4
V, Å^3	1052.40(2)	492.889(15)
T, °C	25	25
M (g/mol)	122.023	122. 635
λ	$CuK_{\alpha1,2}$	$CuK_{\alpha1,2}$

Table S11. Atomic Coordinates of the Structure Refinement of **CF-I**.

atom			**CF-I**		
	x	y	z	B_{iso}	*Occ.*
N1	0.5179(17)	0.8764(9)	0.2701(5)	3.25	1
C2	0.6880(18)	0.7866(16)	0.2469(3)	3.25	1
N3	0.7594(11)	0.6183(14)	0.2713(4)	3.25	1
C4	0.662(2)	0.5319(13)	0.3149(4)	3.25	1
C5	0.487(3)	0.624(3)	0.3370(5)	3.25	1
C6	0.4165(11)	0.794(3)	0.3139(6)	3.25	1
N7	0.7359(9)	0.3660(14)	0.3376(3)	3.25	1
O8	0.7757(7)	0.8701(7)	0.2065(3)	3.25	1
H9	0.467(8)	0.997(7)	0.254(3)	4.0	1
H10	0.669(7)	0.300(8)	0.368(3)	4.0	1
H11	0.852(9)	0.306(8)	0.3218(18)	4.0	1
H12	0.306(6)	0.859(9)	0.326(3)	4.0	1
H13	0.42(5)	0.57(5)	0.365(11)	4.0	0.393(11)
F14	0.387(2)	0.536(2)	0.3805(5)	3.25	0.607(11)

Table S12. Atomic Coordinates of the Structure Refinement of **CF-II**.

atom	**ssF-II**				
	x	y	z	B_{iso}	*Occ.*
N1	-0.069(2)	0.0149(11)	0.2266(10)	3.25	1
C2	-0.182(2)	0.1442(19)	0.2670(6)	3.25	1
N3	-0.097(2)	0.2670(8)	0.2172(7)	3.25	1
C4	0.074(2)	0.2621(11)	0.1279(9)	3.25	1
C5	0.195(3)	0.130(2)	0.0909(10)	3.25	1
C6	0.106(3)	0.0063(14)	0.1379(11)	3.25	1
N7	0.1359(19)	0.3814(8)	0.0737(5)	3.25	1
O8	-0.3389(11)	0.1448(7)	0.3498(5)	3.25	1
H9	-0.118(15)	-0.067(6)	0.260(5)	4.0	1
H10	0.252(13)	0.373(7)	0.015(4)	4.0	1
H11	0.107(13)	0.468(5)	0.104(5)	4.0	1
H12	0.175(13)	-0.086(6)	0.117(4)	4.0	1
H13	0.175(13)	-0.086(6)	0.117(4)	4.0	0.359(9)
F14	0.378(4)	0.130(2)	0.0055(8)	3.25	0.641(9)

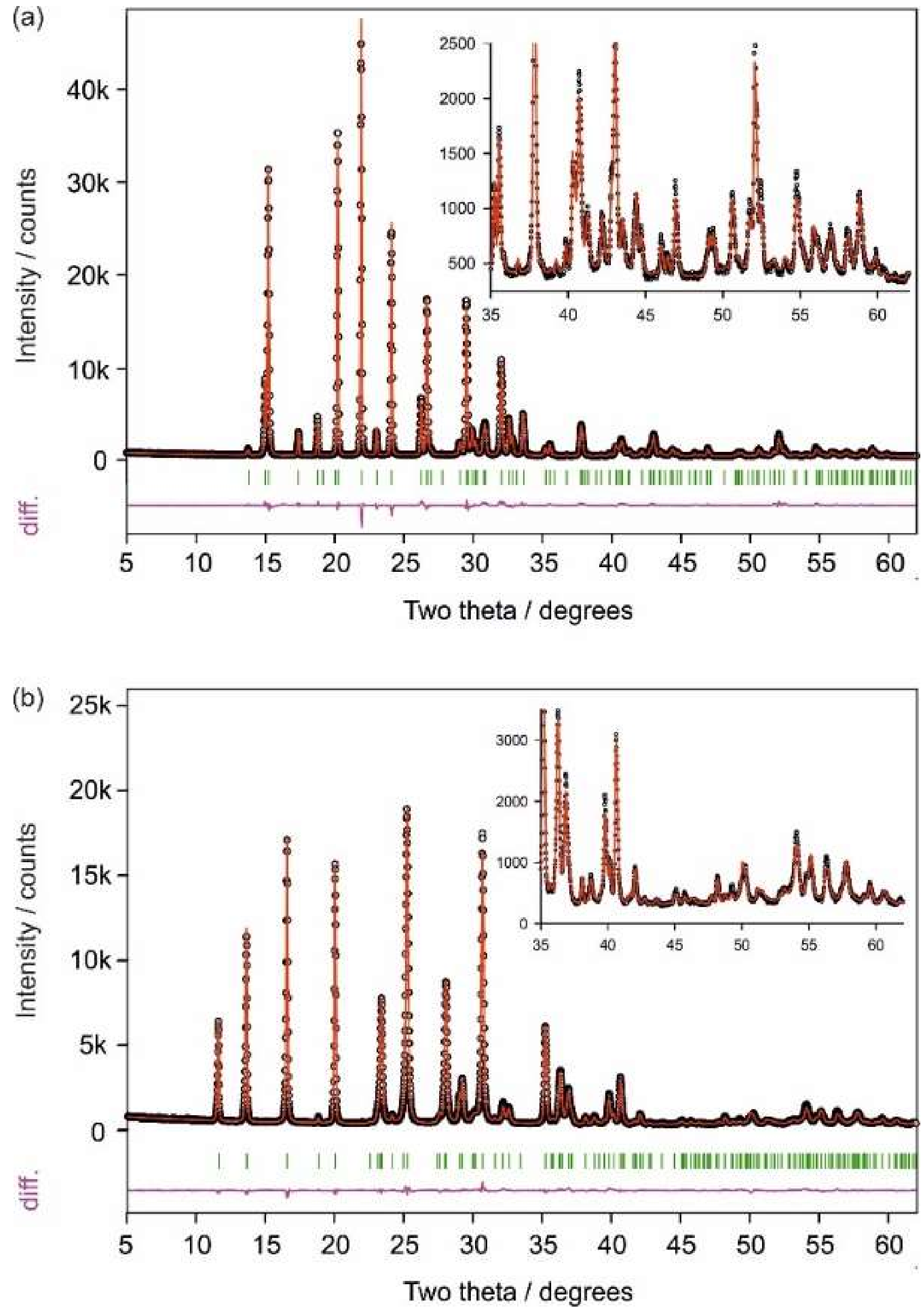

Figure S2. Observed (black points), calculated (red line) and difference (diff.) profiles for the Rietveld refinements of (a) **CF-I** and (b) **CF-II**. Green tick marks denote the peak positions.

2.7. Solvates

2.7.1. Methanol Solvate

Powder X-Ray Diffraction

According to the PXRD patterns (Figure S3) the methanol solvate is not phase pure, but contains traces of the monohydrate. The methanol solvate is isostructural with the 5-flucytosine hemimethanol solvate (MEBQOA[22]).

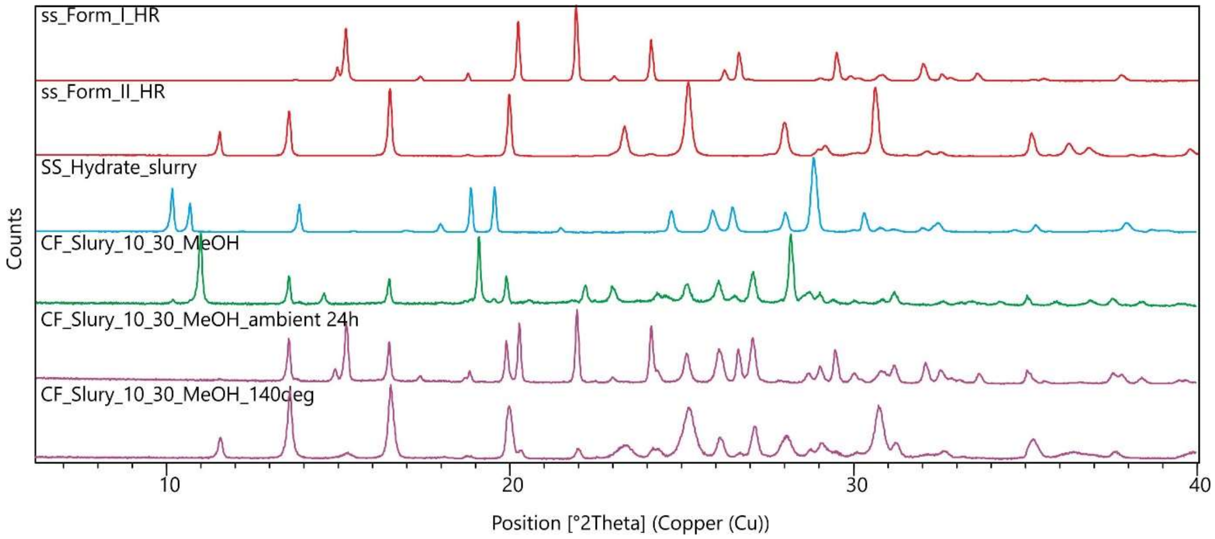

Figure S3. Comparison of anhydrous (red), monohydrate (blue), methanol solvate (green) and desolvated methanol solvate (violet) PXRD patterns. Note that the methanol solvate pattern is not phase pure.

Thermogravimetric Analysis

The methanol solvate loses its solvent molecules immediately when exposed to dry conditions (N_2). The TGA curve shows a two-step mass loss, corresponding to the loss of methanol and water (sample not phase pure). The calculated mass loss for phase pure methanol hemisolvate solid solutions is listen in Table S13.

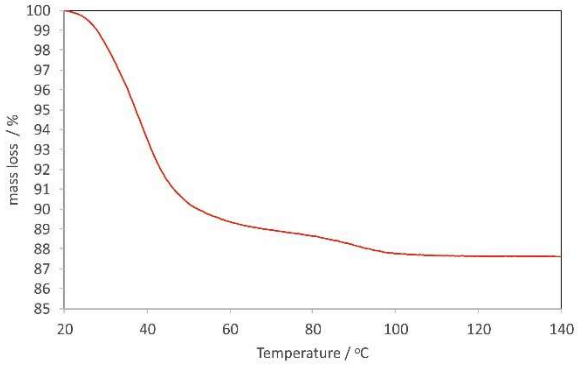

Figure S4. TGA curve of methanol solvate/monohydrate mixture.

Table S13. Solid Solutions of Cytosine and 5-Flucytosine Methanol Hemisolvate.

% 5-Flucytosine	M_r (AH)	W_w[a] / %	W_d[b] / %
100	129.093	11.040	12.410
90	127.294	11.179	12.586
80	125.495	11.321	12.766
70	123.596	11.467	12.952
60	121.897	11.616	13.143
50	120.098	11.770	13.340
40	118.299	11.928	13.543
30	116.500	12.089	13.752
20	114.701	12.256	13.968
10	112.902	12.427	14.190
0	111.103	12.603	14.420

[a]Calculated weight loss relative to wet substance (substance and solvent). [b]Calculated weight loss relative to dry substance (substance without solvent).

2.7.2. Ethanol Solvate

Powder X-Ray Diffraction

The slurry method produced an ethanol solvate with anhydrate II impurities (Figure S5). The PXRD characteristics of the ethanol solvate of the solid solutions suggests that it is isostructural with the 5-flucytosine hemiethanol solvate (see ref. 17).

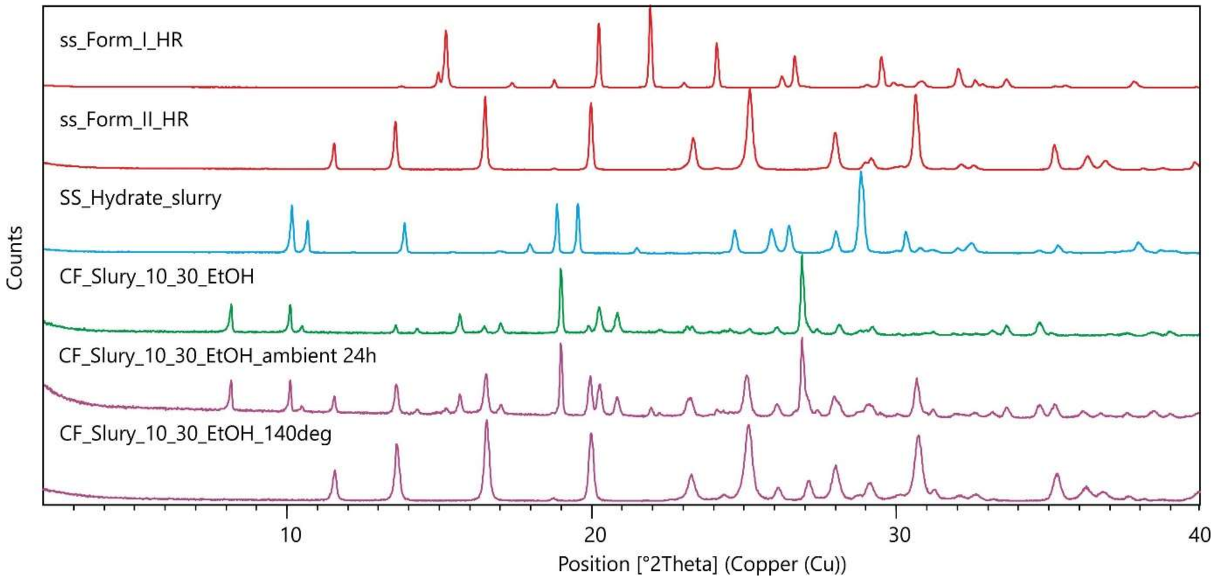

Figure S5. Comparison of anhydrous (red), monohydrate (blue), ethanol solvate (green) and (partly) desolvated ethanol solvate (violet) PXRD patterns. Note that the ethanol solvate patters is not phase pure.

Thermogravimetric Analysis

The ethanol hemisolvate is, compared to the methanol solvate, stable. Desolvation occurs at temperatures > 80 °C. The measured mass loss in the TGA experiments is lower than expected for a hemisolvate (Table S14). This can be related to the fact that the solvate was contaminated with **CF-II**.

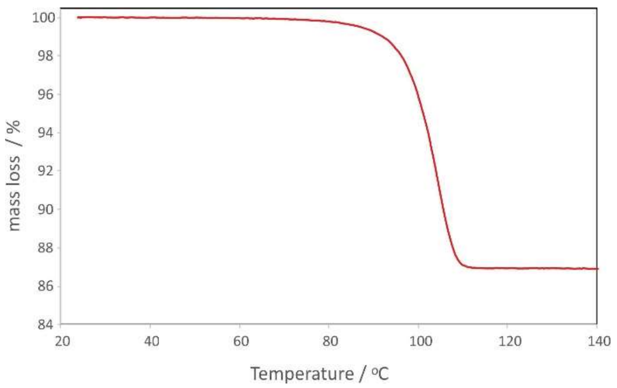

Figure S6. TGA curve of ethanol solvate/**CF-II**.

Table S14. Solid Solutions of Cytosine and 5-Flucytosine Ethanol Hemisolvate.

% 5-Flucytosine	M_r (AH)	Ww[a] / %	Wd[b] / %
100	129.093	15.142	17.843
90	127.294	15.323	18.095
80	125.495	15.508	18.355
70	123.596	15.698	18.622
60	121.897	15.893	18.897
50	120.098	16.093	19.180
40	118.299	16.298	19.471
30	116.500	16.508	19.772
20	114.701	16.724	20.082
10	112.902	16.945	20.402
0	111.103	17.172	20.733

[a]Calculated weight loss relative to wet substance (substance and solvent). [b]Calculated weight loss relative to dry substance (substance without solvent).

2.7.3. Dimethyl Formamide Solvate

Powder X-Ray Diffraction

The PXRD characteristics of the DMF solvate (Figure S7) of the solid solution suggests that it is isostructural with the 5-flucytosine DMF monosolvate (see ref. 17).

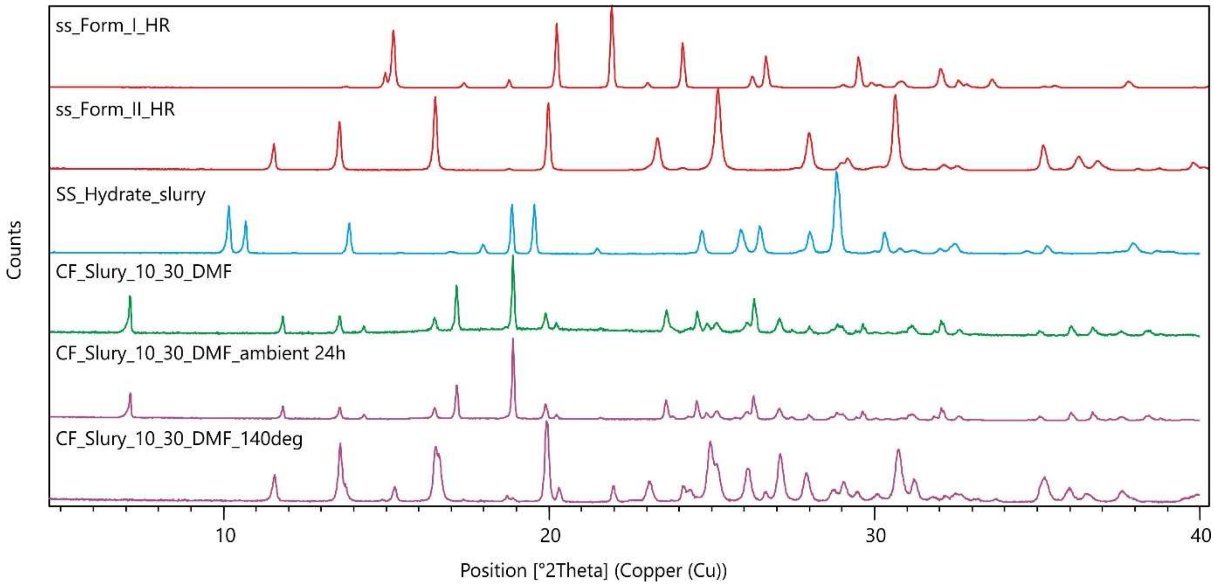

Figure S7. Comparison of anhydrous (red), monohydrate (blue), DMF solvate (green) and PXRD patterns of storage experiments of the DMF solvate (violet).

Thermogravimetric Analysis

The mass loss derived from TGA experiments of the DMF solvate confirms a monosolvate stoichiometry (Figure S8 & Table S15).

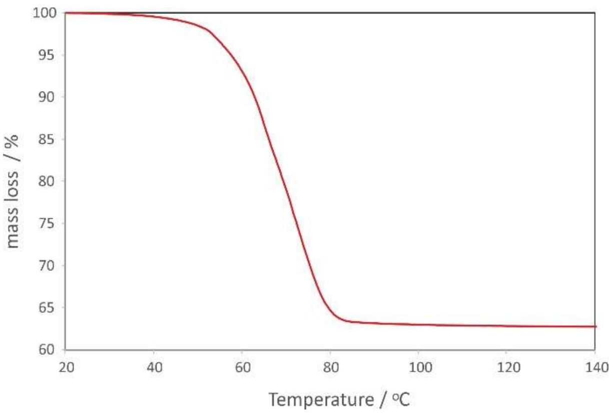

Figure S8. TGA curve of DMF monosolvate.

Table S15. Solid Solutions of Cytosine and 5-Flucytosine DMF Monosolvate.

% 5-Flucytosine	M_r (AH)	Ww[a] / %	Wd[b] / %
100	129.093	36.152	56.622
90	127.294	36.476	57.422
80	125.495	36.807	58.245
70	123.596	37.143	59.092
60	121.897	37.486	59.964
50	120.098	37.835	60.862
40	118.299	38.191	61.788
30	116.500	38.553	62.742
20	114.701	38.922	63.726
10	112.902	39.299	64.742
0	111.103	39.683	65.790

[a]Calculated weight loss relative to wet substance (substance and solvent). [b]Calculated weight loss relative to dry substance (substance without solvent).

2.7.4. Dimethyl Sulfoxide Solvate

The slurry method produced a DMSO solvate with **CF-II** impurities (Figure S9). The PXRD characteristics of the DMSO solvate of the solid solution suggests that it is isostructural with the 5-flucytosine DMSO solvate (DUKWAI[23]).

Powder X-Ray Diffraction

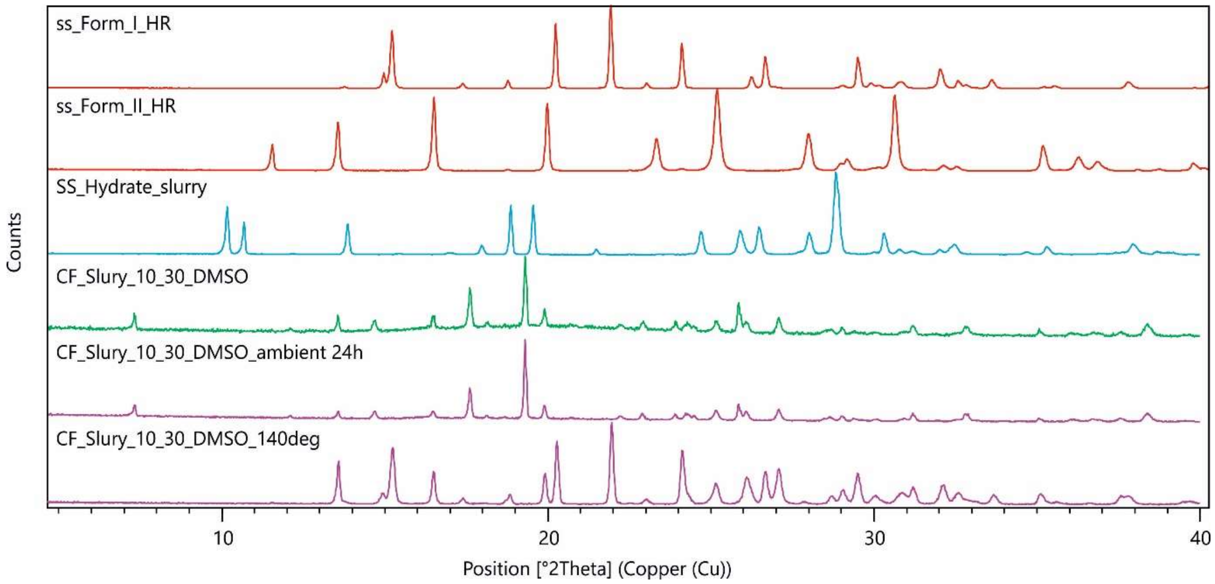

Figure S9. Comparison of anhydrous (red), monohydrate (blue), DMSO solvate (green) and storage experiments of DMSO solvate (violet) PXRD patterns. Note that the DMSO solvate patters is not phase pure.

Thermogravimetric Analysis

The measured mass loss in the TGA experiments is lower than expected for a monosolvate stoichiometry (Table S16), which can be related to the fact that the solvate was not phase pure but contaminated with **CF-II**.

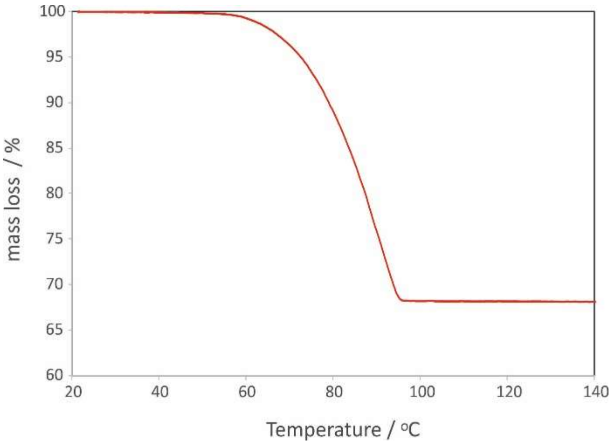

Figure S10. TGA curve of DMSO monosolvate/**CF-II**.

Table S16. Solid Solutions of Cytosine and 5-Flucytosine DMSO Monosolvate.

% 5-Flucytosine	Mr (AH)	Ww[a] / %	Wd[b] / %
100	129.093	37.705	60.526
90	127.294	38.035	61.381
80	125.495	38.371	62.261
70	123.596	38.713	63.167
60	121.897	39.061	64.099
50	120.098	39.416	65.059
40	118.299	39.777	66.049
30	116.500	40.144	67.069
20	114.701	40.519	68.120
10	112.902	40.900	69.206
0	111.103	41.289	70.326

[a] Calculated weight loss relative to wet substance (substance and solvent). [b]Calculated weight loss relative to dry substance (substance without solvent).

2.8. PXRD Comparisons: 5-Flucytosine and Cytosine/5-Flucytosine Solid Solutions

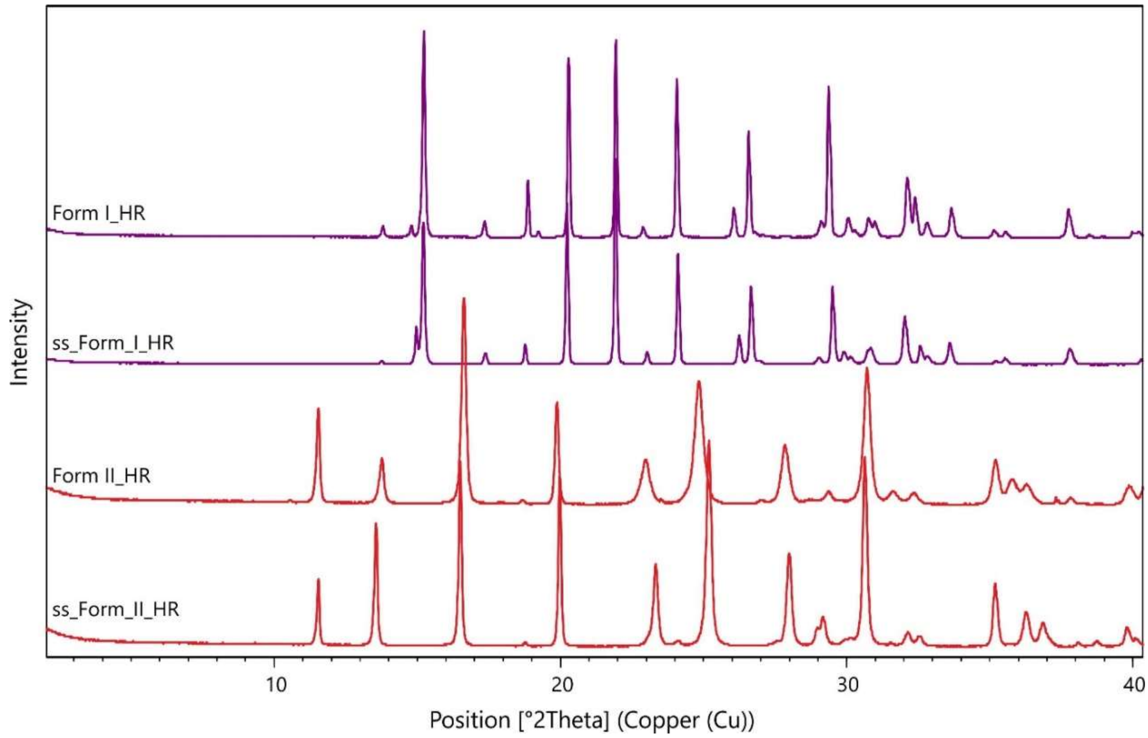

Figure S11. PXRD diffractograms of anhydrous forms of 5-Flucytosine (I and II) and cytosine/5-flucytosine solid solutions (ss).

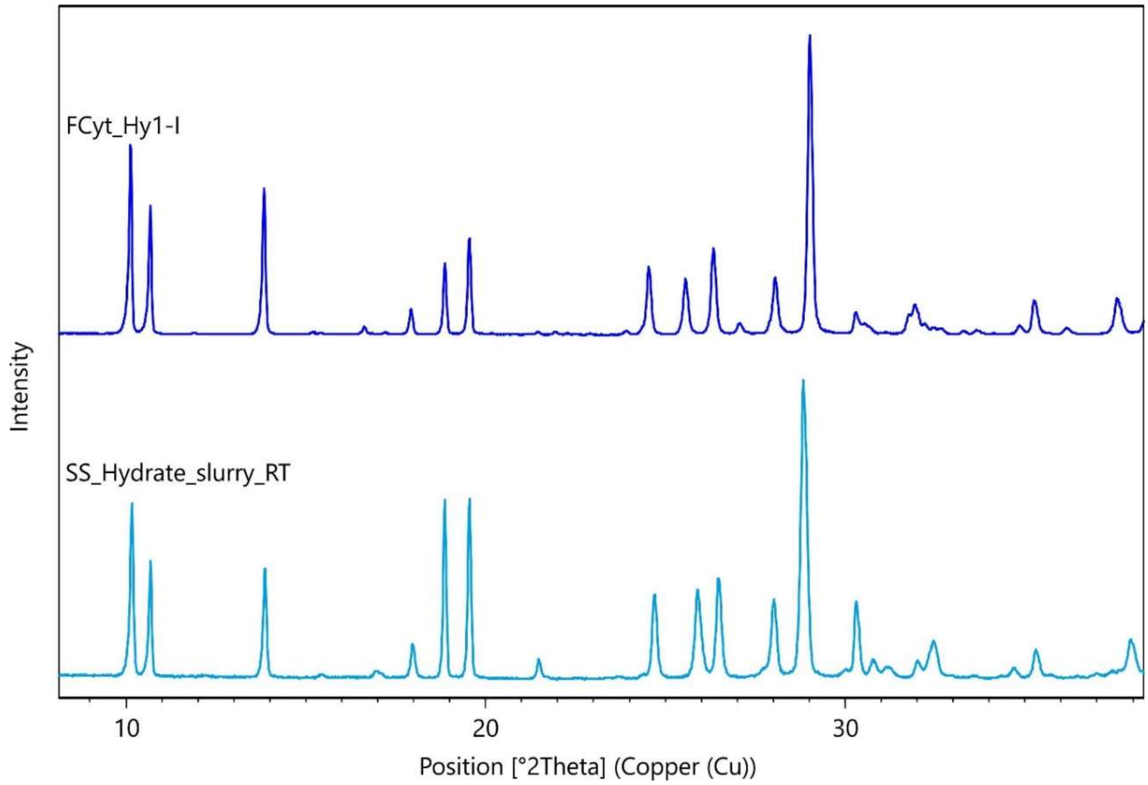

Figure S12. PXRD diffractograms of monohydrate I forms of 5-Flucytosine and cytosine/5-flucytosine solid solution (SS).

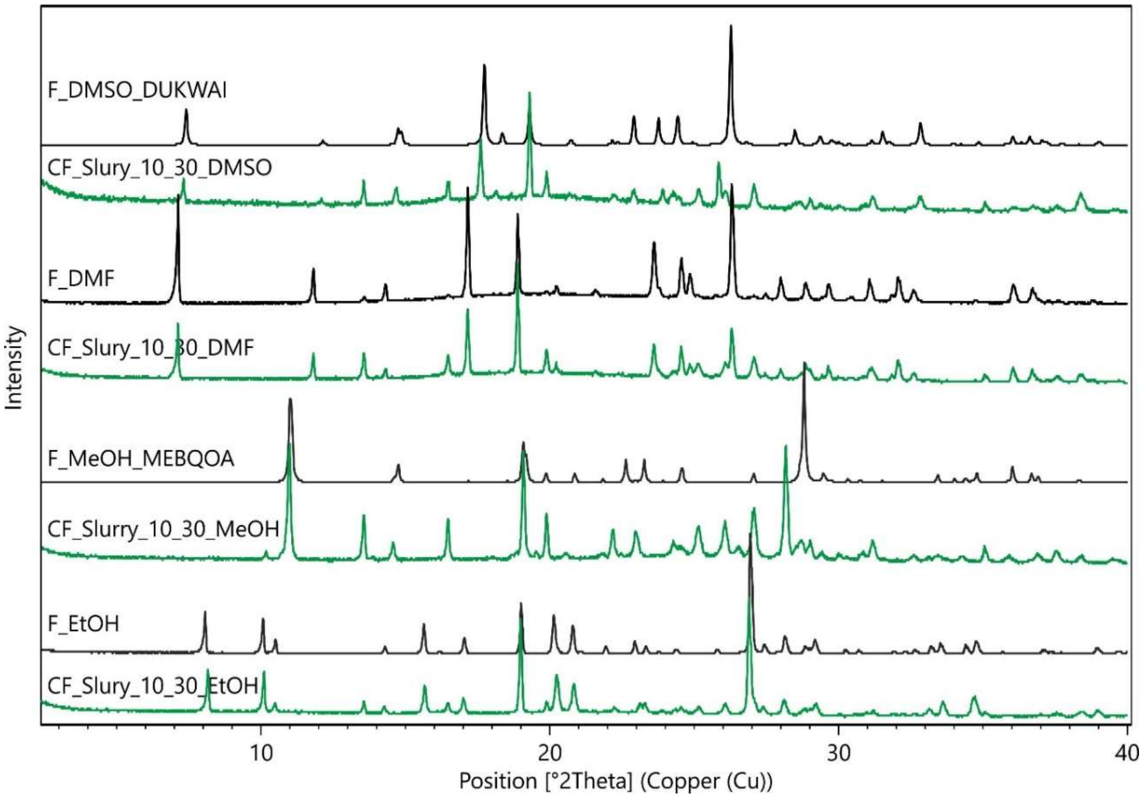

Figure S13. PXRD diffractograms of solvate forms of 5-Flucytosine (F) and cytosine/5-flucytosine solid solutions (CF). Note that the patterns for F_DMSO and F_MeOH were simulated from the single crystal structure data and that some of the experimental solvates are phase mixtures.

3. OVERVIEW SOLID FORMS

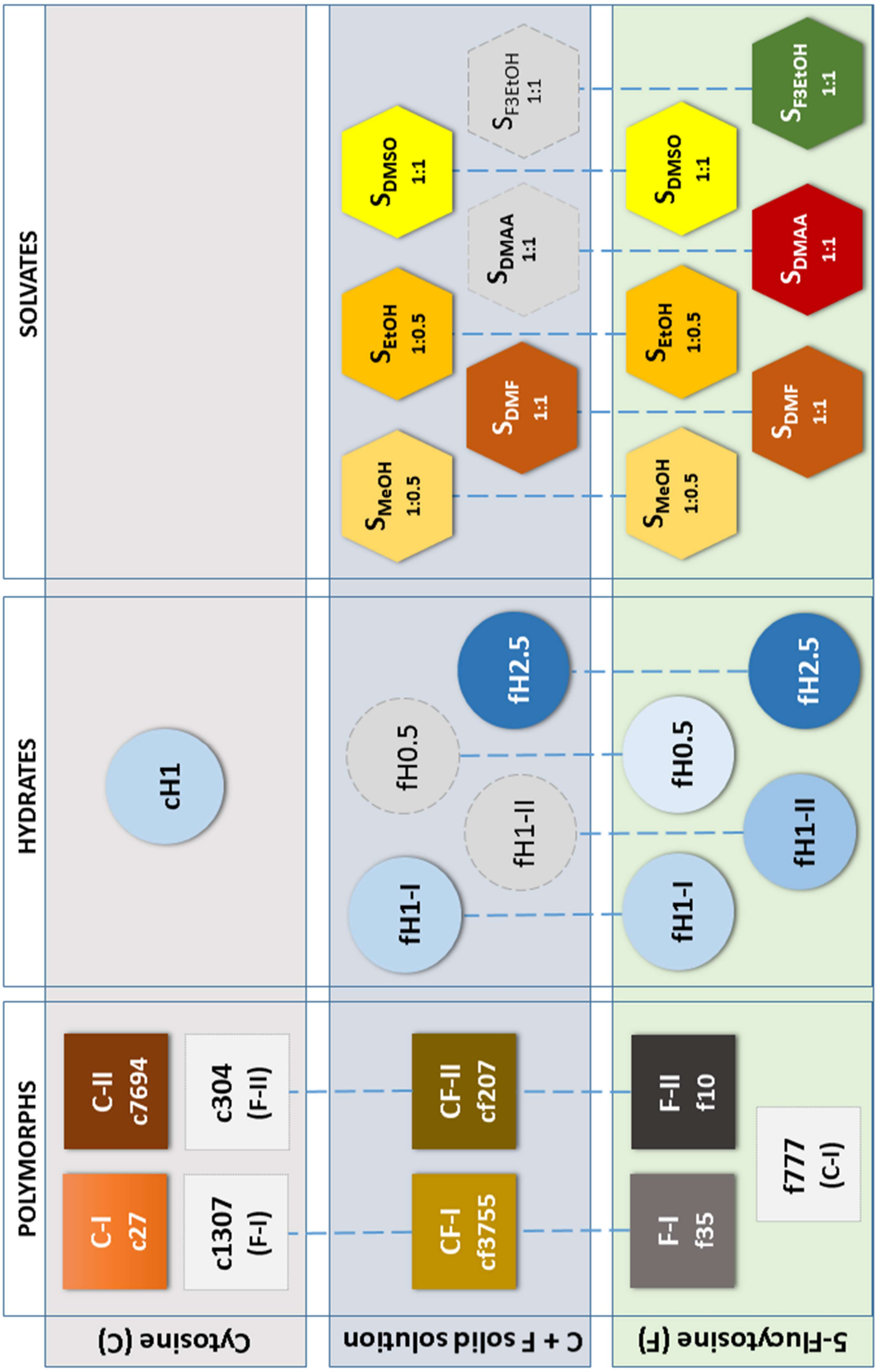

Figure S14. Overview over cytosine (C), 5-flucytosine (F) and mixed solid forms. Isopolymorphs, with the exception of C-I and f777, are connected with blue dashed lines. Structures c1307, c304 and f777 are isopolymorphs that have not been observed yet, but are feasible kinetic forms. Grey symbols indicate hydrates and solvates of the solid solution that are likely to exist as well.

Reference List

1. Groom, C. R.; Bruno, I. J.; Lightfoot, M. P.; Ward, S. C. The Cambridge Structural Database. *Acta Crystallogr. , Sect. B: Struct. Sci. , Cryst. Eng. Mater.* **2016,** *72* (2), 171-179.
2. Karamertzanis, P. G.; Pantelides, C. C. Ab initio crystal structure prediction - I. Rigid molecules. *J. Comput. Chem.* **2005,** *26* (3), 304-324.
3. Karamertzanis, P. G.; Pantelides, C. C. Ab initio crystal structure prediction. II. Flexible molecules. *Molecular Physics* **2007,** *105* (2-3), 273-291.
4. Habgood, M.; Sugden, I. J.; Kazantsev, A. V.; Adjiman, C. S.; Pantelides, C. C. Efficient Handling of Molecular Flexibility in Ab Initio Generation of Crystal Structures. *J. Chem. Theory Comput.* **2015,** *11* (4), 1957-1969.
5. Coombes, D. S.; Price, S. L.; Willock, D. J.; Leslie, M. Role of Electrostatic Interactions in Determining the Crystal Structures of Polar Organic Molecules. A Distributed Multipole Study. *J. Phys. Chem.* **1996,** *100* (18), 7352-7360.
6. Breneman, C. M.; Wiberg, K. B. Determining Atom-Centered Monopoles From Molecular Electrostatic Potentials - The Need For High Sampling Density in Formamide Conformational-Analysis. *J. Comput. Chem.* **1990,** *11* (3), 361-373.
7. Price, S. L.; Leslie, M.; Welch, G. W. A.; Habgood, M.; Price, L. S.; Karamertzanis, P. G.; Day, G. M. Modelling Organic Crystal Structures using Distributed Multipole and Polarizability-Based Model Intermolecular Potentials. *Phys. Chem. Chem. Phys.* **2010,** *12* (30), 8478-8490.
8. Stone, A. J. Distributed multipole analysis: Stability for large basis sets. *J. Chem. Theory Comput.* **2005,** *1* (6), 1128-1132.
9. *GDMA: A Program for Performing Distributed Multipole Analysis of Wave Functions Calculated Using the Gaussian Program System,* version 2.2; University of Cambridge: Cambridge, United Kingdom, 2010
10. Kazantsev, A. V.; Karamertzanis, P. G.; Adjiman, C. S.; Pantelides, C. C. Efficient Handling of Molecular Flexibility in Lattice Energy Minimization of Organic Crystals. *J. Chem. Theory Comput.* **2011,** *7* (6), 1998-2016.
11. Clark, S. J.; Segall, M. D.; Pickard, C. J.; Hasnip, P. J.; Probert, M. J.; Refson, K.; Payne, M. C. First principles methods using CASTEP. *Z. Kristallogr.* **2005,** *220* (5-6), 567-570.
12. Perdew, J. P.; Burke, K.; Ernzerhof, M. Generalized gradient approximation made simple. *Phys. Rev. Lett.* **1996,** *77* (18), 3865-3868.
13. Vanderbilt, D. Soft Self-Consistent Pseudopotentials in a Generalized Eigenvalue Formalism. *Phys. Rev. B* **1990,** *41* (11), 7892-7895.
14. Tkatchenko, A.; Scheffler, M. Accurate Molecular Van Der Waals Interactions from Ground-State Electron Density and Free-Atom Reference Data. *Phys. Rev. Lett.* **2009,** *102* (7), 073005-1-073005/4.
15. Grimme, S. Semiempirical GGA-type density functional constructed with a long-range dispersion correction. *J. Comput. Chem.* **2006,** *27* (15), 1787-1799.
16. *PLATON, A Multipurpose Crystallographic Tool,* Utrecht University: Utrecht, The Netherlands, 2003
17. Braun, D. E.; Kahlenberg, V.; Griesser, U. J. Cyt_FCyt hy. *Mol. Pharm.* **2017,** *submitted*.
18. Chisholm, J. A.; Motherwell, S. COMPACK: a program for identifying crystal structure similarity using distances. *J. Appl. Crystallogr.* **2005,** *38*, 228-231.
19. Markvardsen, A. J.; David, W. I. F.; Johnson, J. C.; Shankland, K. A probabilistic approach to space-group determination from powder diffraction data. *Acta Crystallogr. , Sect. A.* **2001,** *57*, 47-54.
20. David, W. I. F.; Shankland, K.; van de Streek, J.; Pidcock, E.; Motherwell, W. D. S.; Cole, J. C. DASH: a program for crystal structure determination from powder diffraction data. *J. Appl. Crystallogr.* **2006,** *39*, 910-915.
21. *Topas Academic V5,* Coelho Software: Brisbane, 2012
22. Hulme, A. T.; Tocher, D. A. The Discovery of New Crystal Forms of 5-Fluorocytosine Consistent with the Results of Computational Crystal Structure Prediction. *Cryst. Growth Des.* **2006,** *6* (2), 481-487.
23. Tutughamiarso, M.; Bolte, M.; Egert, E. New pseudopolymorphs of 5-fluorocytosine. *Acta Crystallogr. , Sect. C: Cryst. Struct. Commun.* **2009,** *65* (11), o574-o578.
